# Linking big biomedical datasets to modular analysis with Portable Encapsulated Projects

**DOI:** 10.1093/gigascience/giab077

**Published:** 2021-12-06

**Authors:** Nathan C Sheffield, Michał Stolarczyk, Vincent P Reuter, André F Rendeiro

**Affiliations:** Center for Public Health Genomics, University of Virginia, VA 22908, USA; Department of Public Health Sciences, University of Virginia, VA 22908, USA; Department of Biomedical Engineering, University of Virginia, VA 22908, USA; Department of Biochemistry and Molecular Genetics, University of Virginia, VA 22908, USA; Center for Public Health Genomics, University of Virginia, VA 22908, USA; Center for Public Health Genomics, University of Virginia, VA 22908, USA; Genomics and Computational Biology Graduate Group, University of Pennsylvania, PA 19087, USA; Institute for Computational Biomedicine, Weill Cornell Medical College, NY 10021, USA; Caryl and Israel Englander Institute for Precision Medicine, Weill Cornell Medical College, NY 10021, USA

**Keywords:** sample metadata standard, interoperability, sample annotation table, metadata validation schema

## Abstract

**Background:**

Organizing and annotating biological sample data is critical in data-intensive bioinformatics. Unfortunately, metadata formats from a data provider are often incompatible with requirements of a processing tool. There is no broadly accepted standard to organize metadata across biological projects and bioinformatics tools, restricting the portability and reusability of both annotated datasets and analysis software.

**Results:**

To address this, we present the Portable Encapsulated Project (PEP) specification, a formal specification for biological sample metadata structure. The PEP specification accommodates typical features of data-intensive bioinformatics projects with many biological samples. In addition to standardization, the PEP specification provides descriptors and modifiers for project-level and sample-level metadata, which improve portability across both computing environments and data processing tools. PEPs include a schema validator framework, allowing formal definition of required metadata attributes for data analysis broadly. We have implemented packages for reading PEPs in both Python and R to provide a language-agnostic interface for organizing project metadata.

**Conclusions:**

The PEP specification is an important step toward unifying data annotation and processing tools in data-intensive biological research projects. Links to tools and documentation are available at http://pep.databio.org/.

## Introduction

Biological data generation is accelerating, and considerable effort is now being invested in how to best share it. These efforts include expansions of databases [1,2], as well as new data standards, ontologies, and guidelines for data sharing [3–9]. Major effort is being invested in building an open data ecosystem upon which data of many types may be easily shared and reused.

As our ability to generate data increases across scientific disciplines, analysis often becomes the bottleneck of scientific advance. To mitigate this, new computational pipelines are under continuous development. These pipelines are increasingly written using pipeline frameworks, leading to now dozens of such frameworks that simplify developing reusable computational pipelines [10], such as the common workflow language [11], Snakemake [12], Galaxy [13], and Nextflow [14]. Similarly, technological advances such as Linux containers are increasing the portability of computing environments [15–18]. Collectively, these efforts seek to meet the challenge of reproducible analysis in a complicated and growing ecosystem that combines public and private data.

**Figure 1:**
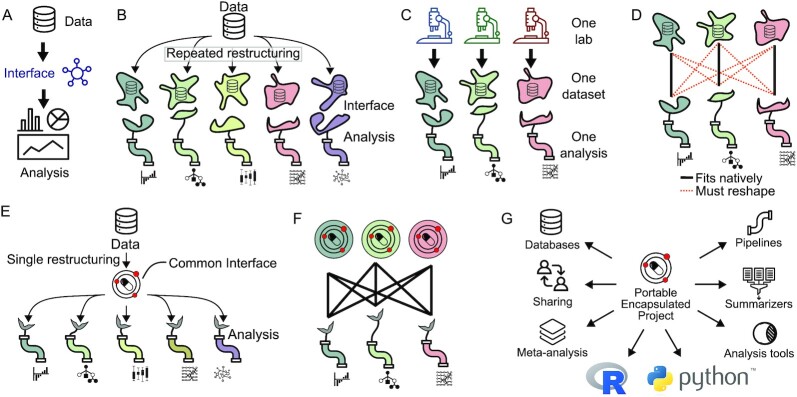
A data interface links data to analysis. (A) Schematic of a data interface. (B) Each analysis typically describes its own unique data interface. (C) The “one lab, one dataset, one analysis” mode of research tightly couples datasets and analysis. (D) With individual data interfaces, running a dataset through multiple analyses requires reshaping the data for every pairwise connection of data and analysis. (E) The PEP specification provides a standardized interface that reduces reshaping. (F) Using PEP, no reshaping is required to run a dataset through a different analytical tool. (G) A PEP may be used in different contexts and by a variety of tools and programming languages.

These dual efforts to (i) curate open biological data and (ii) standardize bioinformatics analysis are certainly complementary. More accessible data combined with easier standardized analysis opens enormous opportunity; however, progress in each area independently does not necessarily make it easier to connect the two. In fact, relatively less effort has been placed at the confluence of data and analysis in biology. We may call this connection a “data interface,” which describes how a dataset connects to an analysis tool (Fig. 1A). As it stands, published bioinformatics pipelines, even if reproducibly built in a standard framework, typically describe a unique data interface. This implies that a user who wants to try multiple pipelines must manually restructure the metadata for each (Fig. 1B). On the flipside, data repositories also typically expose the data in unique ways. In practice, it requires substantial manual effort to plug an arbitrary dataset into an arbitrary analysis tool—even if both adhere to best-practice standards for data sharing or tool development.

This challenge is surmountable for a typical project that links 1 dataset to 1 analysis process—the “one lab, one dataset, one analysis” approach, which has been the dominant model (Fig. 1C). But imagine an attempt to link multiple datasets from multiple sources to multiple analysis tools. Each pair of data and tool requires a unique data description, which probably requires substantial manual data munging (Fig. 1D). The result is that analysis done by an individual laboratory is often restricted to a particular dataset generated by that laboratory for that project. What would it take to build a computing ecosystem that would relax this coupling, making it routine to mix-and-match data and pipelines across groups?

A first step to realize this vision is to standardize the data interface. This would make both datasets and tools more portable, facilitating data integration and tool comparison. To this end, we present the Portable Encapsulated Projects (PEP) specification. The PEP specification standardizes the description of sample-intensive biological research projects, enabling data providers and data users to communicate through a common interface (Fig. 1E). This standardization facilitates using different pipelines for the same datasets, or, equivalently, different datasets for the same pipelines (Fig. 1F).

In addition to standardization, our work with PEP provides 3 additional features that contribute to the interoperability of data: First, the PEP specification provides portability functions called “project modifiers” and “sample modifiers,” which make project metadata annotation independent of a particular computing platform. Second, it provides a validation framework that can be used first to define and then to validate the sample properties required for a particular application. Finally, the project includes tools that read PEPs and handle PEP modifiers in either R or Python, which can be extended by specialized tools.

With these features, PEPs encourage interoperability across data and tools. One result is that PEPs can be re-used for many types of downstream analysis (Fig. 1G). For instance, standardized PEPs enable meta-analysis across hundreds of projects because each project can be read in a uniform way. Instead of requiring a custom project description, file names, and organization, databases could simply provide a schema and use the PEP specifciation to load published projects into a structured database. Tools that summarize processed data can be made to use the same PEP that runs the original workflows, making these summarizing tools more broadly applicable. The PEP specification thus provides a unifying data organization that can be used by many tools to make it easier to share data and tools.

The goal of the PEP specification follows the vision of the Investigation/Study/Assay (ISA) biological metadata management framework [19]. Relative to ISA, the PEP specification emphasizes generality, programmatic metadata preprocessing, and integration into workflow systems. Existing tools can easily accommodate the PEP structure; e.g., Snakemake includes a directive to import a PEP into a workflow that functions alongside earlier, specialized data formats [20]. Similarly, our companion tool, “looper,” can be used to submit arbitrary CWL workflows to a CWL runner for each sample in a PEP. This sets the stage for a single data description that can be used as input for multiple workflows—even workflows built using different frameworks. Example workflows using PEP sample metadata and several different workflow engines are available in the project documentation at http://pep.databio.org/.

## Results

### Basic PEP specification

The PEP specification defines a way to organize project and sample metadata in files using YAML and CSV formats. The term “project” refers to a collection of metadata that describes a set of samples. A “sample” is defined loosely as any unit that can be collected into a project; it consists of sample attributes, usually with 1 or more that point to data files. A “PEP” is a set of files that conform to the PEP specification. A common example could be a typical biological research “project” made up of a set of RNA-sequencing samples grouped to answer a particular question.

**Figure 2:**
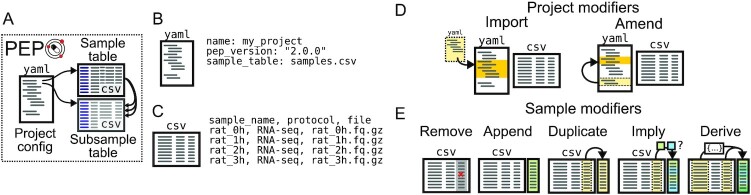
The PEP specification. (A) A PEP consists of a YAML configuration file, a sample table, and a subsample table. (B) The YAML file describes project-level attributes. (C) The sample table (and subsample table) describe sample-level attributes. (D) Project modifiers allow the PEP to import values from other PEPs or to embed multiple variations within a single PEP. (E) Sample modifiers can change sample attributes by using the project config YAML file, without actually changing the CSV file.

The specification defines a PEP in 2 files: an (optional) YAML configuration file and a tabular comma-separated value (CSV) annotation file (Fig. 2A). The configuration file provides project-level descriptions, such as paths to remote or local sources of data, global analysis parameters, or other project attributes. The tabular file is a sample table, providing metadata attributes for each biological specimen included in the project. An optional third file, the subsample table, can be used to specify sample attributes with multiple values (see http://pep.databio.org/ for further details). A basic PEP configuration file has just a few fields in YAML format, such as this example file (Fig.   2B) that points to a “samples.csv” file (Fig. 2C), which contains a header line of sample attributes and then 1 data row per sample. Together, these 2 files describe a minimal project. The basic PEP format is thus extremely flexible and can accommodate assorted sample-intensive biological research project data. Because PEP uses simple plain text files, it is universally accessible, easy to version control, and inexpensive to store.

This simple approach is then extended in 2 critical improvements: first, we added features that improve portability called “project modifiers” and “sample modifiers,” which enable us to remove environment-specific file paths and analysis-specific metadata from the sample table, making it easier to use a single metadata representation for multiple analyses in different computing environments. These “modifiers” are handled by implementations of the PEP specification, which then provide “modified,” or “processed,” sample and project metadata for downstream tools to consume. Second, we built a validation framework for PEPs that includes a base schema to validate generic PEPs along with tools to extend this schema to more specific use cases. This generic + specialization approach allows us to construct re-usable project definitions that can be extended modularly to provide increased specificity. Together, these 2 improvements provide the power and specificity that enable the PEP specification to unify and enhance our metadata descriptions for many types of data-intensive biological research projects. We describe these in more detail below.

### Project modifiers

Project modifiers are special project attributes that provide additional functionality to a project. The 2 modifiers are “import” and “amend,” which allow users to either merge or embed PEPs (Fig. 2D). At times it is useful to create 2 projects that are very similar but differ just in 1 or 2 attributes. For example, you may define a project with 1 set of samples and then want an identical project that uses a different sample table. Or, you may define a project to run on a particular reference genome and want to define a second project that is identical but uses a different reference genome. You could simply define 2 complete PEPs, but this would duplicate information and make it harder to maintain. Instead, project modifiers make it easier to tie projects together through the import and amend relationships.

#### Project modifier: import

The import project modifier allows the configuration file to import other PEPs. The values in the imported files will be overridden by the corresponding entries in the current configuration file. Imports are recursive, so an imported file that imports another file is allowed; the imports are resolved in cascading order with the most distant imports happening first, so the closest configuration options override the more distant ones. Imports provide a way to decouple project settings so that more specific projects can inherit attributes from more general projects. Imports allow users to combine multiple files into 1 PEP description. The import modifier handles sample tables the same way it does any other attribute. If a sample table is specified in both an imported and importing PEP, it does not merge or update individual samples or tables but simply selects the highest priority value of the “sample_table” attribute.

#### Project modifier: amend

The amend project modifier allows the configuration file to embed multiple independent projects within a single PEP. When a PEP is parsed, you may specify 1 or more included amendments, which will amend the values in the processed PEP. Amendments are useful to define multiple similar projects within a single project configuration file. Under the amend key, you specify names of amendments, and then underneath these you specify any project variables that you want to override for that particular amendment. It is also possible to activate multiple amendments in priority order, which allows you to combine different project features on the fly.

Example:

sample_table: annotation.csv

project_modifiers:

amend:

my_project2:

sample_table: annotation2.csv

my_project3:

sample_table: annotation3.csv

When used in tandem, imports and amendments together make it possible to create powerful links between projects and analysis settings that can simplify running multiple analyses across multiple projects.

### Sample modifiers

Sample modifiers are project-level settings that adjust sample attributes. After the sample table is read, sample modifiers are applied, adding new attributes or changing attributes from the original sample table. Sample modifiers enable keeping analysis-specific sample attributes in the project configuration file so the sample table can be more easily shared across projects. This allows the creation of a sample table that does not need to be edited when moved to either a different project or compute environment, making both project and sample metadata more portable.

You can add sample modifiers to a PEP by adding a “sample_modifiers” section to a project configuration file. Within this section, there are 5 subsections corresponding to 5 types of sample modifier (Fig. 2E). Three modifiers—“remove,” “append,” and “duplicate”—are simple operations. The more expressive sample modifiers*—“*imply” and “derive”—lend considerable flexibility to the construction of PEP sample tables.

#### Sample modifier: remove

The remove modifier simply removes a specified attribute from all samples. It can be useful if a particular analysis needs to eliminate a particular attribute without modifying the original sample table.

Example:

sample_modifiers:

remove:

- genome

#### Sample modifier: append

The append modifier adds constant attributes to all samples in a project. For example, if you write “genome: hg38” as an entry under “append”, then when the PEP is parsed, the samples will each have an additional attribute, “genome”, with value “hg38”. This modifier is useful because it allows keeping static attributes in the project configuration file. It also allows you to preserve project-level information (like “genome”) separate from sample-level information, but still pass that information along to pipelines that require it for each sample. This addresses the structural mismatch in independence that follows from project composition—often, samples may be processed independently while having high dependence among their metadata. PEPs are friendly to the “don’t repeat yourself” principle that improves project maintainability.

Example:

sample_modifiers:

append:

genome: hg38

#### Sample modifier: duplicate

The “duplicate” modifier allows copying an existing sample attribute into a new one. For example, the “genome” attribute could be a synonym of the “Genome” attribute. This allows us to tweak settings at the project level, which simplifies use of an alternate pipeline with different requirements, without requiring modification of the underlying sample table that may break earlier analysis. In the key:value pair, the old attribute name listed as key will be duplicated to create a new attribute named with the corresponding value.

Example:

sample_modifiers:

duplicate:

Genome: newattr

#### Sample modifier: imply

The “imply” modifier lets a user add sample attributes that are modulated on the basis of the value of an existing sample attribute. For example, a common use case is to use “imply” to set a “genome” attribute for any sample with a specific value in its “organism” attribute. This enables complete separation of description of sample-intrinsic properties (like organism) from project-level values (like reference genome, which may change).

Example:

sample_modifiers:

imply:

- if:

organism: human

then:

genome_assembly: hg38

#### Sample modifier: derive

The most expressive sample modifier is called “derive.” This modifier allows us to create sample attributes that are derived from other sample attributes. The most common use case is to specify paths to data files at the project level instead of at the sample level. This allows tabular sample descriptions to avoid including any environment-specific information (such as a file path), so that moving a project from one compute environment to another requires editing only a single line in the project configuration file.

The derive modifier consists of 2 pieces of data: First, the “attributes” section lists sample attributes to be derived. Second, the “sources” section contains key-value pairs, where the keys are source names and values are string templates. The source names are the original values of the derived attributes. The string templates are used to derive new attribute values by the PEP processor, replacing the source names in the original table. These templates may contain sample attributes enclosed by curly braces, such as {sample_name}.

Example:

sample_modifiers:

derive:

attributes: [read1, read2]

sources:

key1: "/path/{attr}/{sample_name}.fq"

key2: "/path/{attr}/{sample_name}.fq"

In this example, {attr} and {sample_name} represent other attributes that are present on the sample. These may be populated from the sample table or from other attributes that have been added using a sample modifier such as append.

When derived source paths include a shell variable, derived attributes enable not only a sample table, but an entire PEP, to be made completely portable with no editing. For instance, we could replace “/path” above with “$DATAPATH”, and this PEP would then point to the correct files on any computing environment with the “$DATAPATH” environment variable set.

### Project and sample validation

**Figure 3:**
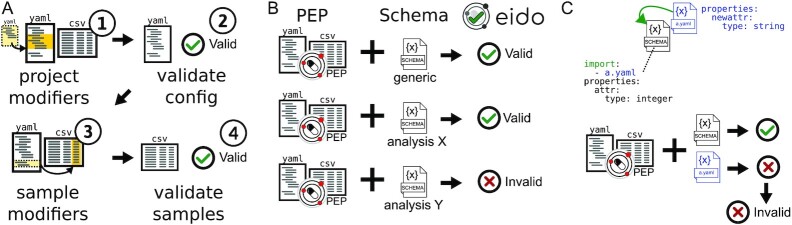
PEPs can be validated against generic or specific schemas. (A) Validation uses 2 steps so samples are validated after PEP modification. (B) A generic schema ensures compliance with the PEP specification, while specialized schemas describe requirements for a particular analysis. (C) PEP schemas can import other schemas.

To make it easier to standardize PEPs across workflows, we also implemented a PEP validation tool called “eido”. Eido is a specialized PEP validator that extends JSON-schema (https://json-schema.org/). Because eido is based on JSON-schema, it inherits explicit variable typing (e.g., string, number, boolean) and restrictions on values (e.g., ranges, regular expressions, enumerated values). Eido then adds a few advanced features that extend the basic vocabulary to tailor it to the PEP use case. For example, our validator adds the term “required_files”, which allows a schema author to indicate which sample attributes must point to files that exist. Eido also adds the ability to validate both project-level and sample-level attributes after PEP project and sample modifier processing. Eido uses a 2-stage validation that first validates the configuration file, and then validates individual samples after they have been processed (Fig. 3A). This ensures that sample attributes that are added or modified can be properly checked. These adjustments to the basic JSON-schema validation allow eido to satisfy the requirements of validating bioinformatics research projects.

Data types are defined using PEP schema files, which may be equivalently saved in either JSON or YAML format. Eido can be used with a generic PEP specification schema to validate a PEP in general. The generic schema defines attributes inherent to the basic PEP specification, including project and sample modifiers, version, and the general data model of project-level and sample-level attributes. A schema for a specific analysis can extend this generic schema, such as by specifying and describing required sample attributes, noting attributes that point to files on disk, and identifying allowable values for given attributes. With this model, tool authors can easily create a schema that describes more specific requirements for a tool, and eido can validate a given PEP to make sure it conforms to both the generic schema and the more stringent schema, ensuring that it can run on a particular tool (Fig. 3B).

For example, an author of a pipeline may write a schema specifying that samples must have attributes named “read1” and “read2”, which must be of type string, and which must point to input files. Furthermore, the schema specifies that samples must have an attribute called “genome” that specifies the genome to align to, perhaps with a list of allowable values. With this schema published, it is now possible to validate a PEP to ensure that it fulfills the requirements for this pipeline. PEP schemas can also import other schemas (Fig. 3C). In this case, the PEP must validate against all requirements specified by imported schemas to be valid. Complete documentation, descriptions of schema features, and example schemas can be found at http://eido.databio.org.

### PEP implementations in R and Python

The reference implementation of the PEP specification is the “peppy” Python package, available from the Python Package Index (PyPI). Peppy instantiates in-memory project objects and provides a Python API for programmatic access to any project metadata from within Python. A user simply creates a Project object with prj = Project("config.yaml") and may now interact with the project metadata within Python. This package is a generic, extensible object framework that enables developers to build additional tools using these objects. For instance, Snakemake relies on the peppy package to handle parsing and reading PEP-formatted project metadata to power a workflow run.

We have also developed an R implementation of PEP in the “pepr” package, available on CRAN. PEPs can be parsed in R with a similar function call, prj = pepr::Project("config.yaml"), which provides an R API for interacting with PEPs in R. These tools provide a PEP project interface to programmers of 2 of the most popular data science programming languages, increasing the portability of PEP projects.

We are interested in future efforts to expand this to other computing frameworks. These APIs provide basic functions for interacting with projects and samples, including setting and accessing variables, extracting the sample attributes and sub-attributes as a tabular object (using pandas in Python and data.table in R), and accessing individual samples as objects. In each case, all the sample and project modifiers are processed behind the scenes so downstream tools can easily make use of the PEP portability features. The formal API is documented in the respective package documentation.

## Discussion

### The promise of PEP

As the amount of available data increases, it is useful to build a common infrastructure to link it to analytical tools. Currently, downloading and analyzing an external dataset requires significant manual investment. Because each analytical pipeline typically has a unique interface to input data, testing multiple competing pipelines on a single dataset requires describing the dataset multiple times. These manual steps hinder re-analysis and re-use of existing data.

We here propose reducing this barrier with the concept of Portable Encapsulated Projects. The PEP specification is at once standardized and flexible. It provides a loose generic specification that can be easily extended for specific use cases. It also provides a validation framework that can easily accommodate both generic and specialized PEPs.

Together, PEPs provide an interface between data and tools that makes each more useful. If a tool developer designs a tool to read PEPs, then it is immediately possible to apply the tool to any published, compliant PEPs. To describe how to use the tool, the developer needs only define a PEP schema, which can be validated using eido; any project defining these attributes would then work without modification. Users then immediately know how to format a project for the tool, and by describing newly generated data in PEP format, they can immediately plug that project into the tool. As developers build pipelines that understand PEP format, they make it simple to apply their pipeline to new PEP-compatible projects as they emerge.

On the flipside, as data producers publish datasets in PEP format, they make it easy for pipeline developers to test new analytical techniques on data from a variety of sources. This will incentivize data sharing and re-use, driving innovation and discovery both in tool development and in understanding of data.

Together, these tools create a programmable link between data and analysis, making it simple to re-analyze an existing dataset with a newly developed pipeline, grab a relevant public dataset to include with newly generated data in a private project, or test a published PEP-compatible pipeline on some in-house data.

### PEP in practice

We and others have successfully used the PEP infrastructure in dozens of projects with hundreds to thousands of individual samples. For example, PEP has been used as the sample metadata structure for ATAC-seq [21] and PRO-seq [22] workflows, which have been used on several downstream projects. We also rely on the PEP parsers for listing reference genome assets for the refgenie server [23, 24]. The PEP specification has been used for a variety of analysis types, such as describing samples for The Cancer Genome Atlas (TCGA) [25], CRISPR-based screening [26], DNA methylation analysis [27], simulated genomic interval data [28], analysis of Type I diabetes genetics [29], and others [30]. A curated list of other research that uses PEP format is maintained in the PEP documentation. These examples and others demonstrate the breadth and versatility of PEP and provide a starting point for interested developers or users who would like to see PEP in action.

### A call for community involvement

To conclude, we offer a call for community involvement to support reaching the vision of metadata interoperability. Three key steps will be required before this can happen: First, we need tools that support and extend the PEP specification; second, we need adoption by workflow engines; and finally, we need support of public datasets and data repositories to accept and provide data interoperable with the PEP specification.

A first step will be to build tools that operate in this area. To facilitate community uptake, we are developing a series of tools that subscribe to the PEP standard. Herein, we described Python and R packages that read PEPs, along with eido for PEP validation. These core tools can form the foundation of new tools, and we hope that others in the community will use them to add functionality to the PEP ecosystem. For our needs, we are extending these capabilities with several ongoing projects: First, “geofetch” is a data fetcher that accepts a list of SRA or Gene Expression Omnibus (GEO) accession numbers and then downloads raw sequence data from the SRA and constructs a PEP, ready to be plugged into a PEP-compatible analysis tool. Second, “looper” is a workflow-engine–agnostic command submission engine that reads PEP-formatted sample data and runs arbitrary commands. Finally, “BiocProject” is an upcoming project that adds bioconductor-specific functionality to PEPs, simplifying biological data analysis of PEPs in R.

A second step will be for workflow engines to adopt the PEP specification for sample metadata. Workflow engines are becoming a critical component of biological data analysis, and as such, they provide an important incentive for the way users and tool developers organize metadata. Unfortunately, most workflow engines still require a custom format for describing input metadata. We have been reaching out to workflow engine communities, such as the Snakemake [12] and CWL [11] communities, which already have some support for PEP-formatted metadata. We are also developing a metadata conversion function in eido that would allow users to write custom formatters, making it easier to fit PEP-formatted metadata into custom analyses. We invite collaboration and involvement from other workflow-oriented communities who could support a community effort for standardized metadata organization that spans workflow engines.

And third, another important step will be the creation of datasets and data repositories that understand this format, for both submission and download. We encourage authors of individual papers to consider using a PEP-structured sample table when publishing descriptions for individual projects. And we invite large-scale data providers to make it possible to download data descriptions in PEP-compatible files, and even to submit data in PEP-valid format.

To our knowledge, this is the first major effort to produce a universal specification and framework for collections of biological sample metadata geared toward metadata and data processing. PEPs can be tailored with ease to specific use cases with schemas that define specific tool requirements. We anticipate that these tools will encourage both bioinformatics pipeline developers and data producers to subscribe to a common format, benefiting both and leading to increased ability to extract useful information from biological data.

## Availability of Supporting Source Code and Requirements

The formal PEP specification can be found at http://pep.databio.org/. Software mentioned is available in the following locations:

**Project name: eido**

Project home page: http://eido.databio.org/

Operating system: Platform independent

Programming language: Python

License: BSD-2

RRID: SCR_021076

biotools: eido-python-package

**Project name: peppy**

Project home page: http://peppy.databio.org/

Operating system: Platform independent

Programming language: Python

License: BSD-2

RRID: SCR_021078

biotools: peppy-python-package

**Project name: pepr**

Project home page: http://pepr.databio.org/

Operating system: Platform independent

Programming language: R

License: BSD-2

RRID: SCR_021077

biotools: pepr-R-package

## Supplementary Material

giab077_GIGA-D-20-00319_Original_Submission

giab077_GIGA-D-20-00319_Revision_1

giab077_GIGA-D-20-00319_Revision_2

giab077_GIGA-D-20-00319_Revision_3

giab077_Response_to_Reviewer_Comments_Original_Submission

giab077_Response_to_Reviewer_Comments_Revision_1

giab077_Response_to_Reviewer_Comments_Revision_2

giab077_Reviewer_1_Report_Original_Submission Daniel NÃ¼st, Dipl-Geoinf. -- 1/8/2021 Reviewed

giab077_Reviewer_2_Report_Original_Submission Katy Wolstencroft -- 1/18/2021 Reviewed

giab077_Reviewer_2_Report_Revision_1 Katy Wolstencroft -- 6/7/2021 Reviewed

## Data Availability

Snapshots of the repositories are available in the *GigaScience* GigaDB repository [31].

## Abbreviations

API: Application Programming Interface; ATAC-seq: Assay for Transposase-Accessible Chromatin with high-throughput sequencing; CWL: Common Workflow Language; ISA: Investigation/Study/Assay; JSON: Javascript Object Notation; PEP: Portable Encapsulated Project; PRO-seq: Precision Run-On Sequencing; SRA: Sequence Read Archive.
