# Supplementary material for: Linking big biomedical datasets to modular analysis with Portable Encapsulated Projects
Source: Gigascience. 2021 Dec 6;10(12):giab077. doi: 10.1093/gigascience/giab077 (PMC8673555; doi:10.1093/gigascience/giab077)
Supplement: giab077_GIGA-D-20-00319_Revision_1 [file giab077_giga-d-20-00319_revision_1.pdf]

GigaScience

Linking big biomedical datasets to modular analysis with Portable Encapsulated Projects

--Manuscript Draft--

**Manuscript Number:**	GIGA-D-20-00319R1
**Full Title:**	Linking big biomedical datasets to modular analysis with Portable Encapsulated Projects
**Article Type:**	Technical Note
**Funding Information:**	National Institute of General Medical Sciences (R35GM128636) — Dr Nathan C Sheffield
**Abstract:**	Organizing and annotating biological sample data is critical in data-intensive bioinformatics. Unfortunately, incompatibility is common between metadata format of a data source and that required by a processing tool. There is no broadly accepted standard to organize metadata across biological projects and bioinformatics tools, restricting the portability and reusability of both annotated datasets and analysis software. To address this, we present Portable Encapsulated Projects (PEP), a formal specification for biological sample metadata structure. The PEP specification accommodates typical features of data-intensive bioinformatics projects with many samples, whether from individual experiments, organisms, or single cells. In addition to standardization, the PEP specification provides descriptors and modifiers for different organizational layers of a project, which improve portability among computing environments and facilitate use of different processing tools. PEP includes a schema validator framework, allowing formal definition of required metadata attributes for any type of biomedical data analysis. We have implemented packages for reading PEPs in both Python and R to provide a language-agnostic interface for organizing project metadata. PEP therefore presents an important step toward unifying data annotation and processing tools in data-intensive biological research projects.
**Corresponding Author:**	Nathan C. Sheffield<br><br>UNITED STATES
**Corresponding Author Secondary Information:**	
**Corresponding Author's Institution:**	
**Corresponding Author's Secondary Institution:**	
**First Author:**	Nathan C Sheffield
**First Author Secondary Information:**	
**Order of Authors:**	Nathan C Sheffield
	Michal Stolarczyk
	Vincent P Reuter
	Andre Rendeiro
**Order of Authors Secondary Information:**	
**Response to Reviewers:**	Response to review uploaded as a PDF for formatting.
**Additional Information:**	
**Question**	**Response**
Are you submitting this manuscript to a special series or article collection?	No

Powered by Editorial Manager® and ProduXion Manager® from Aries Systems Corporation

**Experimental design and statistics**<br><br>Full details of the experimental design and statistical methods used should be given in the Methods section, as detailed in our Minimum Standards Reporting Checklist. Information essential to interpreting the data presented should be made available in the figure legends.<br><br>Have you included all the information requested in your manuscript?	Yes
**Resources**<br><br>A description of all resources used, including antibodies, cell lines, animals and software tools, with enough information to allow them to be uniquely identified, should be included in the Methods section. Authors are strongly encouraged to cite Research Resource Identifiers (RRIDs) for antibodies, model organisms and tools, where possible.<br><br>Have you included the information requested as detailed in our Minimum Standards Reporting Checklist?	Yes
**Availability of data and materials**<br><br>All datasets and code on which the conclusions of the paper rely must be either included in your submission or deposited in publicly available repositories (where available and ethically appropriate), referencing such data using a unique identifier in the references and in the "Availability of Data and Materials" section of your manuscript.<br><br>Have you have met the above requirement as detailed in our Minimum Standards Reporting Checklist?	Yes

Manuscript

Click here to access/download;Manuscript;manuscript.pdf

RESEARCH ARTICLE

Linking big biomedical datasets to modular analysis with Portable Encapsulated Projects

Nathan C. Sheffield[1,2,3,4,✉], Michał Stolarczyk[1], Vincent P. Reuter[1,5], and André F. Rendeiro[6,7]

[1]Center for Public Health Genomics, University of Virginia
[2]Department of Public Health Sciences, University of Virginia
[3]Department of Biomedical Engineering, University of Virginia
[4]Department of Biochemistry and Molecular Genetics, University of Virginia
[5]Genomics and Computational Biology Graduate Group, University of Pennsylvania
[6]Institute for Computational Biomedicine, Weill Cornell Medical College
[7]Caryl and Israel Englander Institute for Precision Medicine, Weill Cornell Medical College
✉ Correspondence: nsheffield@virginia.edu

Organizing and annotating biological sample data is critical in data-intensive bioinformatics. Unfortunately, metadata formats from a data provider are often incompatible with requirements of a processing tool. There is no broadly accepted standard to organize metadata across biological projects and bioinformatics tools, restricting the portability and reusability of both annotated datasets and analysis software. To address this, we present Portable Encapsulated Projects (PEP), a formal specification for biological sample metadata structure. The PEP specification accommodates typical features of data-intensive bioinformatics projects with many samples, whether from individual experiments, organisms, or single cells. In addition to standardization, the PEP specification provides descriptors and modifiers for different organizational layers of a project, which improve portability among computing environments and facilitate use of different processing tools. PEP includes a schema validator framework, allowing formal definition of required metadata attributes for any type of biomedical data analysis. We have implemented packages for reading PEPs in both Python and R to provide a language-agnostic interface for organizing project metadata. PEP therefore presents an important step toward unifying data annotation and processing tools in data-intensive biological research projects.

Introduction

Biological data generation is accelerating, and considerable effort is now being invested in how to best share it. These efforts include expansions of databases[1,2] as well as new data standards and ontologies, including the FAIR guiding principles and other guidelines for data sharing[3–8]. Major effort is being invested in building an open data ecosystem upon which data of many types may be easily shared and reused.

As our ability to measure and store data has increased across scientific disciplines, analysis has frequently become the bottleneck of scientific advance. To mitigate this, new computational pipelines and analysis approaches are under constant development. These pipelines are increasingly federated though pipeline frameworks, leading to now dozens of such frameworks that simplify developing reusable computational pipelines[9], as well as standards for workflows such as the common workflow language[10], SnakeMake[11], Galaxy[12], and Nextflow[13]. Similarly, new containerization technology is making computing environments more portable [[14];;[15];[16]] and efforts to build data commons[17] and cloud analysis platforms[18] are bringing analysis to data hosted in the cloud. Collectively, these efforts seek to meet the challenge of reproducible analysis in a complicated and growing ecosystem that combines public and private data.

Efforts to both curate open biological data and to standardize bioinformatics analysis are certainly complementary, but progress in each area independently does not necessarily make it easier to connect the two. In fact, relatively less effort has been placed at the confluence of data and analysis in biology. We may call this connection a "data interface," which describes how a dataset connects to an analysis tool (Fig. 1A). As it stands, published bioinformatics pipelines, even if reproducibly built in a standard framework, typically describe a unique data interface, requiring a user to manually structure data repeatedly to fit each pipeline (Fig. 1B). On the flipside, data repositories also typically expose an individual procedure such as an API for accessing the data. In practice, it requires substantial manual effort to plug an arbitrary dataset into an arbitrary analysis tool – even if both adhere to best-practice community sharing and analysis development standards.

This challenge is surmountable for a typical project that links one data set to one analysis process – the *one lab, one dataset, one analysis* approach, which has been the dominant model (Fig. 1C). But imagine an attempt to link multiple datasets from multiple sources to multi-

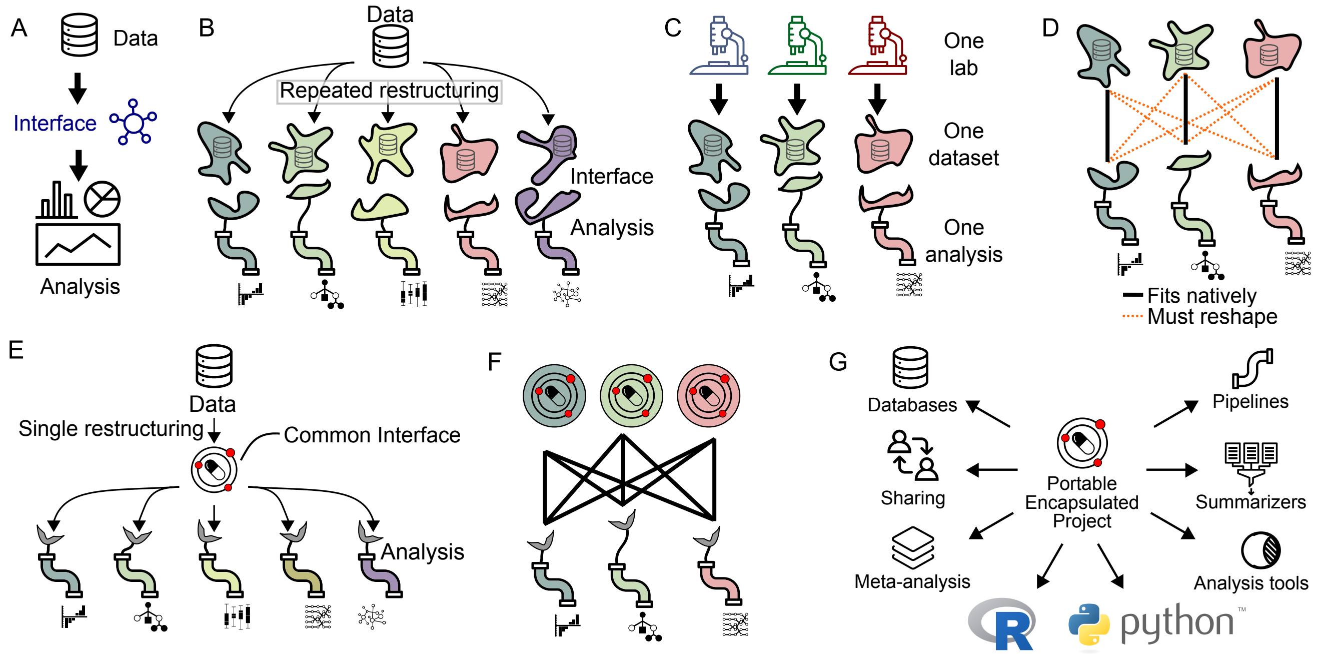

***Fig. 1**: **A data interface links data to analysis.** A) Schematic of a data interface. B) Each analysis typically describes its own unique data interface. C) The* one lab, one dataset, one analysis *mode of research tightly couples datasets and analysis. D) With individual data interfaces, running a data set through multiple analyses requires reshaping the data for every pairwise connection of data and analysis. E) The PEP specification provides a standardized interface that reduces reshaping. F) Using PEP, no reshaping is required to run a data set through a different analytical tool. G) A PEP may be used in different contexts, and by a variety of tools and programming languages.*

ple analysis tools. Each pair of data and tool requires a unique data description, which probably requires substantial manual data munging (Fig. 1D). The result is that analysis done by an individual lab is often restricted to a particular dataset generated by that lab for that project. What would it take to build a computing ecosystem that would relax this coupling, making it routine to mix-and-match data and pipelines across groups?

A first step to realize this vision is to standardize the data interface. This would make both datasets and tools more portable, facilitating data integration and tool comparison. To this end, we present the Portable Encapsulated Projects (PEP) specification. The PEP specification standardizes the description of sample-intensive biological research projects, enabling data providers and data users to communicate through a common interface (Fig. 1E). This standardization facilitates using different pipelines for the same datasets (Fig. 1F). In addition to standardization, the PEP specification provides powerful portability called *project modifiers* and *sample modifiers* that make project metadata annotation independent of a particular computing platform. PEP also provides a customizable validation framework that can be used to first define and then to validate the sample properties required for a particular application. Finally, we provide tools that read PEPs and handle PEP modifiers in R and Python, which can be extended by specialized tools.

PEP thus provides a unifying data organization that can be employed by many tools to make it easier to share data and tools. The goal of PEP follows the vision of the Investigation/Study/Assay (ISA) biological metadata management framework[19]. Relative to ISA, PEP emphasizes generality, programmatic metadata preprocessing, and integration into workflow systems. Existing tools can easily accommodate the PEP structure; for example, SnakeMake includes a special directive to directly import a PEP into a workflow that functions alongside earlier, specialized data formats. Similarly, our companion tool, *looper*, can be used to submit arbitrary CWL workflows to a CWL runner for each sample in a PEP project. This sets the stage for a single data description that can be used as input for multiple workflows – even workflows built using different frameworks.

Together, these advantages realize a unified specification that can be read and processed by many types of downstream analysis (Fig. 1G). By standardizing the description of project metadata and providing standalone, modular tools to read that standard, we simplify existing processes and enable new types of analysis. For instance, standardized PEPs enables meta-analysis that assesses sample properties across hundreds of projects, since each project can be read the same way. Databases, instead of requiring a custom project description and file naming and organization could instead simply provide a schema and use PEP to load published projects into a structured database. Tools that summarize processed data can be made to use the same PEP that runs the original workflows, making these kind of summarizing tools more broadly applicable. And finally, a shared metadata structure simplifies sharing across individuals and tools.

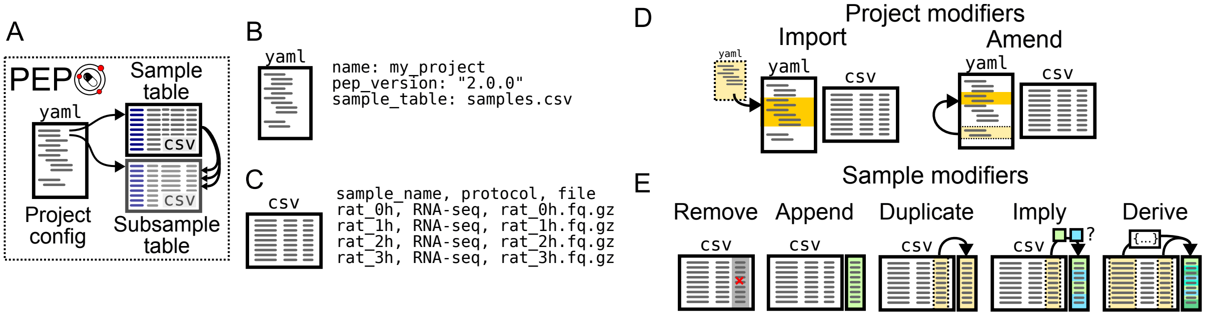

Fig. 2: The PEP specification. *A) A PEP consists of a YAML configuration file, a sample table, and a subsample table. B) The YAML file describes project-level attributes. C) The sample table (and subsample table) describe sample-level attributes. D) Project modifiers allow the PEP to import values from other PEPs, or embed multiple variations within a single PEP. E) Sample modifiers can change sample attributes by using the project config YAML file, without actually changing the CSV file.*

Results

Basic PEP specification

The *PEP specification* defines a way to organize project and sample metadata in files using `YAML` and `CSV` formats. The term *project* refers to a collection of metadata that describes a set of samples. A *sample* is defined loosely as any unit that can be collected into a project; it consists of sample attributes, usually with one or more that point to data files. A *PEP* is a set of files that conform to the PEP specification. An common example could be a typical biological research *project* made up of a set of RNA-seq *samples* grouped to answer a particular question.

The specification defines a PEP in two files: A `YAML` configuration file, and a tabular comma-separated value (`CSV`) annotation file (Fig. 2A). The configuration file provides project-level descriptions, such as paths to remote or local sources of data, global analysis parameters, or other project attributes. The tabular file is a sample table, providing metadata attributes for each biological specimen included in the project. An optional third file, the subsample table, can be used to specify sample attributes with multiple values (see http://pep.databio.org for further details). A basic PEP configuration file has just a few fields in `YAML` format, such as this example `YAML` file (Fig. 2B) that points to a `samples.csv` file (Fig. 2C), which contains a header line of sample attributes and then one data row per sample. Together, these two files describe a minimal project. The basic PEP format is thus extremely flexible and can accommodate assorted sample-intensive biological research project data. Because PEP uses simple plain text files, it is universally accessible, easy to version control, and inexpensive to store.

This very simple approach is then extended in two critical improvements: First, we added features that improve portability called *project modifiers* and *sample modifiers*, which enable us to remove environment-specific file paths and analysis-specific metadata from the sample table, making it easier to use a single metadata representation for multiple analyses in different computing environments. These *modifiers* are handled by implementations of the PEP specification, which then provide *modified*, or *processed*, sample and project metadata for downstream tools to Consume. Second, we built a validation framework for PEPs that includes a base schema to validate generic PEPs along with tools to extend this schema to more specific use cases. This generic + specialization approach allows us to construct a re-usable project definitions that can be extended modularly to provide increased specificity. Together, these two improvements provide the power and specificity that enables PEP to unify and enhance our metadata descriptions for many types of data-intensive biological research projects. We describe these in more detail below.

Project modifiers

Project modifiers are special project attributes that provide additional functionality to a project. The two modifiers are *import* and *amend*, which allow users to either merge or embed PEPs (Fig. 2D). At times it is useful to create two projects that are very similar, but differ just in one or two attributes. For example, you may define a project with one set of samples, and then want an identical project that uses a different sample table. Or, you may define a project to run on a particular reference genome, and want to define a second project that is identical, but uses a different reference genome. You could simply define 2 complete PEPs, but this would duplicate information and make it harder to maintain. Instead, project modifiers make it easier to tie projects together through the *import* and *amend* relationships.

Project modifier: import

The *import* project modifier allows the configuration file to import other PEPs. The values in the imported files will be overridden by the corresponding entries in the current configuration file. Imports are recursive, so an imported file that imports another file is allowed; the imports are resolved in cascading order with the most distant imports happening first, so the closest configuration options override the more distant ones. Imports

provide a way to decouple project settings so that more specific projects can inherit attributes from more general projects. Imports allow users to combine multiple files into one PEP description. The import modifier handles sample tables the same way it does any other attribute. If a sample table is specified in both an imported and importing PEP, it does not merge or update individual samples or tables, but simply selects the highest priority value of the `sample_table` attribute.

Project modifier: amend

The *amend* project modifier allows the configuration file to embed multiple independent projects within a single PEP. When a PEP is parsed, you may specify one or more included amendments, which will amend the values in the processed PEP. Amendments are useful to define multiple similar projects within a single project configuration file. Under the *amend* key, you specify names of amendments, and then underneath these you specify any project variables that you want to override for that particular amendment. It is also possible to activate more than one amendment in priority order, which allows you to combine different project features on-the-fly.

Example:

```
sample_table: annotation.csv
project_modifiers:
  amend:
    my_project2:
      sample_table: annotation2.csv
    my_project3:
      sample_table: annotation3.csv
```

When used in tandem, imports and amendments together make it possible to create powerful links between projects and analysis settings that can simplify running multiple analyses across multiple projects.

Sample modifiers

Sample modifiers are project-level settings that adjust sample attributes. After the sample table is read, sample modifiers are applied, adding new attributes or changing attributes from the original sample table. Sample modifiers enable keeping analysis-specific sample attributes in the project configuration file so the sample table can be more easily shared across projects. This allows the creation of a sample table that does not need to be edited when moved to either a different project or compute environment, making both project and sample metadata more portable.

You can add sample modifiers to a PEP by adding a `sample_modifiers` section to a project configuration file. Within this section, there are 5 subsections corresponding to 5 types of sample modifier (Fig. 2E). Three modifiers – *remove*, *append*, and *duplicate* – are very simple operations. The more expressive sample modifiers – *imply* and *derive* – lend considerable flexibility to the construction of PEP sample tables.

Sample modifier: remove

The *remove* modifier simply removes a specified attribute from all samples. It can be useful if a particular analysis needs to eliminate a particular attribute without modifying the original sample table.

```
sample_modifiers:
  remove:
    - genome
```

Sample modifier: append

The *append* modifier adds constant attributes to all samples in a project. For example, if you write `genome: hg38` as an entry under `append`, then when the PEP is parsed, the samples will each have an additional attribute, `genome`, with value `hg38`. This modifier is useful because it allows keeping static attributes in the project configuration file. It also allows you to preserve project-level information (like `genome`) separate from sample-level information, but still pass that information along to pipelines that require it for each sample. This addresses the structural mismatch in independence that follows from project composition – very often, samples may be processed independently while having high dependence among their metadata. PEPs are friendly to the *don't repeat yourself* principle that improves project maintainability.

Example:

```
sample_modifiers:
  append:
    genome: hg38
```

Sample modifier: duplicate

The *duplicate* modifier allows copying an existing sample attribute into a new one. For example, the "genome" attribute could be a synonym of the "Genome" attribute. This allows us to tweak settings at the project level, which simplifies use of an alternate pipeline with different requirements, without requiring modification of the underlying sample table that may break earlier analysis. In the `key:value` pair, the old attribute name listed as `key` will be duplicated to create a new attribute named with the corresponding `value`.

Example:

```
sample_modifiers:
  duplicate:
    Genome: newattr
```

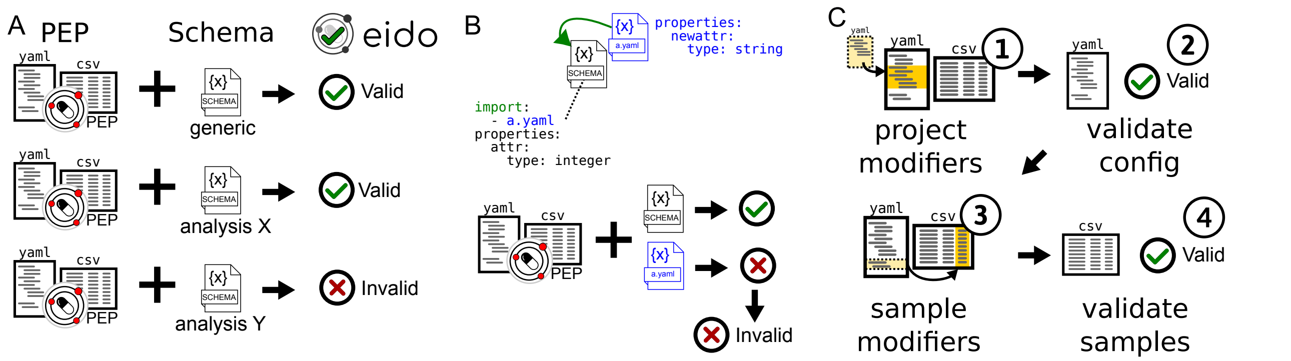

*Fig. 3: **PEPs can be validated against generic or specific schemas.** A) A generic schema ensures compliance with the PEP specification, while specialized schemas describe requirements for a particular analysis. B) PEP schemas can import other schemas. C) Validation uses two steps so samples are validated* after *PEP modification.*

Sample modifier: imply

The *imply* modifier lets a user add sample attributes that are modulated based on the value of an existing sample attribute. For example, a common use case is to use *imply* to set a *genome* attribute for any sample with a specific value in its *organism* attribute. This enables complete separation of description of sample-intrinsic properties (like organism) from project-level values (like reference genome, which may change).

Example:

```
sample_modifiers:
  imply:
    - if:
        organism: "human"
      then:
        genome_assembly: "hg38"
```

Sample modifier: derive

The most expressive sample modifier is called *derive*. This modifier allows us to create sample attributes that are derived from other sample attributes. The most common use case is to specify paths to data files at the project level instead of at the sample level. This allows tabular sample descriptions to avoid including any environment-specific information (such as a file path), so that moving a project from one compute environment to another requires editing only a single line in the project configuration file.

The *derive* modifier consists of two pieces of data: First, the *attributes* section lists sample attributes to be derived. Second, the *sources* section contains key-value pairs, where the keys are source names and values are string templates. The source names are the original values of the derived attributes. The string templates are used to derive new attribute values by the PEP processor, replacing the source names in the original table. These templates may contain sample attributes enclosed by curly braces, such as {`sample_name`}.

Example:

```
sample_modifiers:
  derive:
    attributes: [read1, read2]
    sources:
      key1: "/path/{attr}/{sample_name}.fq"
      key2: "/path/{attr}/{sample_name}.fq"
```

In this example, {`attr`} and {`sample_name`} represent other attributes that are present on the sample. These may be populated from the sample table, or from other attributes that have been added using a sample modifier such as `append`.

When derived source paths include a shell variable, derived attributes enable not only a sample table, but an entire PEP, to be made completely portable with no editing. For instance, we could replace `/path/` above with `$DATAPATH`, and this PEP would then point to the correct files on any computing environment with the `$DATAPATH` environment variable set.

Project and sample validation

To make it easier to build valid PEPs, we also implemented a PEP validation tool called `eido`. Eido is a specialized PEP validator based on JSON-schema (https://json-schema.org/). Complete documentation, descriptions of schema features, and example schemas can be found at eido.databio.org. Schema files may be equivalently saved in either JSON or YAML format. Eido can be used with a generic PEP specification schema to validate a PEP in general. Even more important, tool authors can provide a schema that describes more specific requirements for a tool, and eido can validate a given PEP to make sure it conforms to both the generic schema and the more stringent schema, ensuring that it can run on a particular tool (Fig. 3A).

For example, an author of a pipeline may write a schema specifying that samples must have attributes named `read1` and `read2`, which must be of type `string`, and which must point to input files. Furthermore, the schema specifies that samples must have an attribute called `genome` that specifies the genome to align to,

perhaps with a list of allowable values. With this schema published, it is now possible to validate a PEP to ensure that it fulfills the requirements for this pipeline. PEP schemas can also import other schemas (Fig. 3B). In this case, the PEP must validate against all requirements specified by imported schemas to be valid.

Specific schemas for PEPs are written using JSON-schema with a few additions that extend the basic vocabulary to tailor it to the PEP use case. For example, our validator adds the term `required_files`, which allows a schema author to indicate which sample attributes must point to files that exist. Because eido is based on JSON-schema, it inherits the explicit variable typing (*e.g.* `string`, `number`, `boolean`), and restrictions on values (*e.g.* ranges, regular expressions, enumerated values). Eido uses a two-stage validation that first validates the configuration file, and then validates individual samples *after* they have been processed (Fig. 3C). This ensures that sample attributes that are added or modified can be properly checked. These adjustments to the basic JSON-schema validation allow eido to satisfy the requirements of validating bioinformatics research projects.

PEP implementations in R and Python

The reference implementation of the PEP specification is the `peppy` python package, available from the Python Package Index (PyPI). Peppy instantiates in-memory project objects and provides a Python API for programmatic access to any project metadata from within Python. A user simply creates a Project object (`prj = Project("config.yaml")`) and may now interact with the project metadata within Python. This package is a generic, extensible object framework that enables developers to build additional tools using these objects. For instance, SnakeMake relies on the `peppy` package to handle parsing and reading PEP-formatted project metadata to power a workflow run.

We have also developed an R implementation of PEP in the `pepr` package, available on CRAN. PEP files can be parsed in R with a similar function call, `prj = pepr::Project("config.yaml")`, which provides an R API for interacting with PEPs in R. These tools provide a PEP project interface to programmers of two of the most popular data science programming languages, increasing portability of PEP projects. We and others have successfully used this infrastructure in dozens of projects with hundreds to thousands of individual samples.

We are interested in future efforts to expand this to other computing frameworks. These APIs provide basic functions for interacting with projects and samples, including setting and accessing variables, extracting the sample attributes and sub-attributes as a tabular object (using pandas in Python and data.table in R), accessing individual samples as objects. In each case, all the sample and project modifiers are processed behind the scenes so downstream tools can easily make use of the PEP portability features. The formal API is documented in the respective package documentation.

Discussion

As the amount of available data increases, it is useful to build a common infrastructure to link it to analytical tools. Currently, downloading and analyzing an external dataset requires significant manual investment. Because each analytical pipeline typically has a unique interface to input data, testing multiple competing pipelines on a single dataset requires describing the dataset multiple times. These manual steps hinder re-analysis and re-use of existing data.

We here propose reducing this barrier with the concept of Portable Encapsulated Projects. The PEP specification is at once standardized and flexible. It provides a loose generic specification that can be easily extended for specific use cases. It also provides a validation framework that can easily accommodate both generic and specialized PEPs.

Together, PEP provides an interface between data and tools that makes each more useful. If a tool developer designs a tool to read PEPs, then it is immediately possible to apply the tool to any published, compliant PEPs. To describe how to use the tool, the developer needs only define a PEP schema, which can be validated using `eido`; any project defining these attributes would then work without modification. Users then immediately know how to format a project for the tool, and by describing newly generated data in PEP format, they may immediately plug that project into the tool. As developers build pipelines that understand PEP format, they make it simple to apply their pipeline to new PEP-compatible projects as they emerge.

On the flipside, as data producers publish datasets in PEP format, they make it easy for pipeline developers to test new analytical techniques on data from a variety of sources. This will incentivize data sharing and re-use, driving innovation and discovery both in tool development and in understanding of data.

Together, these tools create a programmable link between data and analysis, making it simple to re-analyze an existing dataset with a newly developed pipeline, grab a relevant public dataset to include with newly generated data in a private project, or test a published PEP-compatible pipeline on some in-house data.

To conclude, we offer a call for community involvement to support reaching the vision of metadata interoperability. Three key steps will be required before this can happen: First, we need tools that support and extend the PEP specification; second, we need adoption by workflow engines; and finally, we need support of public

datasets and data repositories to accept and provide data that fits the system.

A first step will be to build tools that operate in this area. To facilitate community uptake, we are developing a series of tools that subscribe to the PEP standard. Above, we described Python and R packages that read PEPs, along with `eido` for PEP validation. These core tools can form the foundation of new tools, and we hope that others in the community will use them to add functionality to the PEP ecosystem. For our needs, we are extending these capabilities with several ongoing projects: First, `geofetch` is a data fetcher that accepts a list of SRA or GEO accession numbers and then downloads raw sequence data from the Sequence Read Archive and constructs a PEP, ready to be plugged into a PEP-compatible analysis tool. Second, `looper` is a workflow-engine-agnostic command submission engine that reads PEP-formatted sample data and runs arbitrary commands. Finally, `BiocProject` is an upcoming project that adds bioconductor-specific functionality to PEPs, simplifying biological data analysis of PEPs in R.

A second step will be for workflow engines to adopt PEP as a way to specify samples. Workflow engines are becoming a critical component of biological data analysis, and as such, they provide an important incentive for the way users and tool developers organize metadata. Unfortunately, most workflow engines still require a custom format for describing input metadata. We have been reaching out to workflow engine communities, such as the SnakeMake[10] and CWL[11] communities, which already have some support for PEP-formatted metadata. We are also working on a conversion function in `eido` that would allow users to write custom formatters, making it easier to fit PEP-formatted metadata into custom analyses. We invite collaboration and involvement from other workflow-oriented communities who could support a community effort for standardized metadata organization that spans workflow engines.

And third, another important step will be for datasets and data repositories that understand this format, both for submission and download. We encourage authors of individual papers to consider using a PEP-structured sample table when publishing descriptions for individual projects. And we invite large-scale data providers to make it possible to download data descriptions in PEP-compatible files, and even to submit data in PEP-valid format.

To our knowledge, this is the first major effort to produce a universal specification and framework for collections of biological sample metadata geared toward metadata and data processing. PEP can be tailored with ease to specific use cases with schemas that define specific tool requirements. We anticipate that these tools will encourage both bioinformatics pipeline developers and data producers to subscribe to a common format, benefiting both and leading to increased ability to extract useful information from biological data.

Availability

All described software is BSD2-licensed and developed on GitHub at github.com/pepkit. The Python implementation is on PyPI and the R implementation is on CRAN. The formal PEP specification can be found at pep.databio.org.

Identifiers:

- `eido`: RRID:SCR_021076; biotools:eido-python-package
- `pepr`: RRID:SCR_021077; biotools:pepr-R-package
- `peppy`: RRID:SCR_021078; biotools:peppy-python-package

Acknowledgments

We thank Johannes Köster, Jason Smith, Aaron Gu, and the Sheffield lab for input. This work is funded by the National Institutes of Health Institute for General Medical Sciences (NIGMS) award R35GM128636 to NCS.

References

1. Barrett, T. *et al.* NCBI GEO: Archive for functional genomics data sets–update. *Nucleic Acids Res.* **41**, D991–D995 (2013).

2. Leinonen, R., Sugawara, H., Shumway, M. & Collaboration, I. N. S. D. The sequence read archive. *Nucleic Acids Res.* **39**, D19–D21 (2011).

3. Hoehndorf, R., Slater, L., Schofield, P. N. & Gkoutos, G. V. Aber-OWL: A framework for ontology-based data access in biology. *BMC Bioinformatics* **16**, 26 (2015).

4. Malladi, V. S. *et al.* Ontology application and use at the ENCODE DCC. *Database* **2015**, (2015).

5. Wilkinson, M. D. *et al.* The FAIR guiding principles for scientific data management and stewardship. *Sci. Data* **3**, 160018 (2016).

6. Birney, E., Vamathevan, J. & Goodhand, P. Genomics in healthcare: GA4GH looks to 2022. (2017) doi:10.1101/203554.

7. Krumholz, H. M. & Waldstreicher, J. The yale open data access (YODA) project–a mechanism for data sharing. *The New England journal of medicine* **375**, 403–405 (2016).

8. Jupp, S. *et al.* The EBI RDF platform: Linked open data for the life sciences. *Bioinformatics* **30**, 1338–1339 (2014).

9. Leipzig, J. A review of bioinformatic pipeline frameworks. *Brief Bioinform* (2016) doi:10.1093/bib/bbw020.

10. Amstutz, P. *et al.* Common workflow language, v1.0. (2016) doi:10.6084/m9.figshare.3115156.v2.

11. Köster, J. & Rahmann, S. Snakemake–a scalable bioinformatics workflow engine. *Bioinformatics* **28**, 2520–2522 (2012).

12. Afgan, E. *et al.* The galaxy platform for accessible, reproducible and collaborative biomedical analyses: 2016 update. *Nucleic Acids Research* **44**, W3–W10 (2016).

13. Ewels, P. A. *et al.* The nf-core framework for community-curated bioinformatics pipelines. *Nature Biotechnology* **38**, 276–278 (2020).

14. Merkel, D. Docker: Lightweight linux containers for consistent development and deployment. *Linux Journal* **2014**, 2 (2014).

15. Kurtzer, G. M., Sochat, V. & Bauer, M. W. Singularity: Scientific containers for mobility of compute. *PLOS ONE* **12**, e0177459 (2017).

16. Sheffield, N. C. Bulker: A multi-container environment manager. *OSF Preprints* (2019) doi:10.31219/osf.io/natsj.

17. Volchenboum, S. L. *et al.* Data commons to support pediatric cancer research. *American Society of Clinical Oncology Educational Book* **37**, 746–752 (2017).

18. Fenstermacher, D. *et al.* The cancer biomedical informatics grid (caBIG). *Conference proceedings : ... Annual International Conference of the IEEE Engineering in Medicine and Biology Society. IEEE Engineering in Medicine and Biology Society. Annual Conference* **1**, 743–746 (2005).

19. Rocca-Serra, P. *et al.* ISA software suite: Supporting standards-compliant experimental annotation and enabling curation at the community level. *Bioinformatics* **26**, 2354–2356 (2010).

Personal Cover Click here to access/download;Personal Cover;response.pdf

April 20, 2021

Dear Editor,

Thank you for the opportunity to revise our manuscript. We would like to resubmit our manuscript for publication in *GigaScience*. We appreciate the careful review and constructive suggestions provided by the reviewers. We have addressed all reviewer concerns, and the manuscript is substantially improved after making the suggested changes. Our point-by-point response to reviewers follows.

We hope the reviewers will view our response positively and welcome any additional feedback as we seek to bring this work to a wider audience.

Sincerely,

Nathan Sheffield

Nathan Sheffield, PhD, on behalf of all co-authors
Assistant Professor, Center for Public Health Genomics, University of Virginia
www.databio.org
434-924-8278

Reviewer #1:

The manuscript at hand presents the PEP specification and corresponding tools. It is well written and understandable and I have no comments on the form or structure. The visual presentation of the concepts in Figs. 1-3 is excellent. The simplistic specification suppoted by open tools are very good and presented very well. The modifier approach is novel to me and indeed powerful. Thank you for a well-written article and the opportunity to get to know a very interesting collection of tools. I have no expertise in biological data, but could follow the text and successfully tried your tools, also thanks to the extensive online documentation. The presented software seems to be vetted very well through previous usage in real scientific workflows and connected to other relevant tools (CWL, Snakemake) instead of reinventing the wheel.

The article requires minor revisions (no additional experiments or implementations).

> *Wow, thank you for the very positive comments. We really appreciate you taking the time to thoroughly investigate our work. We have spent many years developing the software and documentation behind this idea so we appreciate that you have recognized the utility. We hope you can find a way to make this useful in your own work!*

Introduction

1. "Data generation has outpaced analysis, making analysis . . . " - I stumbled over this a bit, a new notion for me, maybe you can provide a reference?

   We clarified the sentence: "As our ability to measure and store data has increased across scientific disciplines, analysis has frequently become the bottleneck of scientific advance"

2. The colours of "Fits natively" and "Must reshape" were hard to distinguish at first glance - maybe use both colours and different dashes?

   Thank you, revised as suggested.

3. What is PEP's relation to common format specifications for scientific data from other domains? https://www.researchobject.org/ or eLife's ERA come to mind, though they have a larger scope and thereby are probably harder to establish than your more focused approach. IMO worth discussing, but not critical.

We've been working with the Research Objects group loosely for awhile. Research Objects have now been supplanted by their next iteration of the idea, which is called RO-Crate, the difference being that Research Objects was based on complicated ontologies, whereas RO-Crate is simplified and now based on schema.org. In general the research object/RO-Crate concept more generic concept than PEP. They are also, for the moment at least, mostly focused on packaging up workflows. PEP takes a workflow-agnostic approach, and is more specialized on a specific schema for sample metadata. RO doesn't really provide formats and is just sort of more flexible and nebulous. For example, a PEP can be included as an element in a Research Object. I have reached out to Stian, the leader of that project, again now to touch base once more.

ERA is a bit of a different beast, again with more focus on actual execution and publication of code. PEP is totally agnostic to execution of downstream analysis, it's just about how your organize your sample metadata in a way that is standardized. So, you could use PEP as the way to organize and read your metadata within a code block of an ERA chunk, like with any code block.

4. The presented Python and R tools seem to be only for reading PEPs. Can you please clarify what you mean with "tools that read and process PEPs" - I think I'm missing an example how then data is loaded from the PEP and some analysis actually happens. If this is to extensive to include, maybe reference one in the various online documentations of the tools.

   Sorry for the confusion – I guess the word 'process' is overloaded here. By 'process', we were referring to the project and sample modifiers. So, the project configuration file provides some powerful ways to adjust the sample metadata. The R and Python packages implement this functionality, returning to the user a set of "processed samples" – here, "processed" just means the sample and project attributes have been populated according to the PEP configuration settings – not *"running the data through a workflow". This is the job of the workflow manager/framework or code, to which PEP is completely agnostic. This is what SnakeMake or CWL would do, for example. Snakemake, for example, loads the PEP using peppy, which "processes" the PEP to create populated sample objects, and then proceeds to* really *process the same in the workflow. The decoupling of "reading/populating" the sample objects, and then "running the workflow" is one of the primary purposes of PEP. We've revised the sentence to clarify this distinction to: "tools that read PEPs and handle PEP modifiers".*

5. Fig. 1G seems to be not fully explained. I understand this subfigure casts a wide net, but I'd still suggest to have a paragraph explaining your understanding or examples of these possibilities.

   - How are PEPs useful for meta-analysis? What are "summarizers"?
   - How are the connected to databases?
   - No.4 above may serve as an example for "Analysis tools"?

   Added the following paragraph to clarify this vision: Together, these advantages realize a unified specification that can be read and processed by many types of downstream analysis (Fig. 1G). By standardizing the description of project metadata and providing standalone, modular tools to read that standard, we enable meta-analysis that assesses sample properties across hundreds of projects, since each project can be easily read the same way. Databases, instead of requiring a custom project description and file naming and organization, could instead simply provide a schema and use PEP to load published projects into a structured database.

Analysis tools, like workflows or more specialized software, could easily read this same format, so that the process of submitting a finished project to a database requires little manual effort. Tools that summarize processed data can be made to use the same PEP that runs the original workflows, making these kind of summarizing tools more broadly applicable. And finally, a shared metadata structure simplifies sharing projects across individuals, tools, and computing environments.

Results

1. I think the decision for YAML and CSV as file formats and the provisiong of Python and R libraries to use PEPs is great (suitability of the chosen methods).

2. However, the rationales could be explained a bit more in the text, e.g., plain text formats are great for versioning and preserveration, but also have their limits; R + Python represent XX percent of the used tools (with reference).

   Thanks for the suggestion, we have added a sentence to discuss this. I'm not sure how to quantify the R + Python use though, since it seems so domain-specific and broad. I added a note about expanding to other languages as a future consideration. We wrote R and Python packages because that's what we use for our work; there's a java implementation in the works, but that's not my group's specialty. So, I'm hopeful that if others find PEP useful and need another language, it's not too hard to make an equivalent core functionality that can run the modifiers in whatever language is needed. My goal was for the specification to be standalone, apart for particular implementations.

   "Because PEP uses simple plain text files, it is universally accessible, easy to version control, and inexpensive to store.";

3. Data citation (giving credit) is an important part of open science practice. Do you plan to explicitly support or model these kinds of relations in you configuration files, especially with project modifiers and re-combination of PEPs?

   This is a good idea that we hadn't really considered. I think it would be make sense as an extension section in a PEP, with a standalone tool, that would formalize the citation. I have no direct response right now, as this is not implemented yet, but I have created a new issue to start to think about the way to proceed on this. You can follow along or chime in here if you like: https://github.com/pepkit/pepspec/issues/13

4. Is it possible to add arbitrary new fields to the configuration as a tool or user, or will it make it invalid?

   PEP itself imposes no restrictions here, so yes, arbitrary new fields can be added and used. The restrictions come in the form of a JSON-schema – so, for a particular use case, someone can define the allowable fields and exclude things if they want. But by default with JSON schema, everything is valid unless explicitly restricted.

5. Page 3 "see documentation for further details" would be more accessible with a footnote pointing to the meant docs.

   Updated to URL: http://pep.databio.org

6. Re. the contents of a PEP: "such as paths to sources of data" - Can you please clarify is this includes remote data sources? Might be worth putting into the discussion, if you think that could be a worthwhile extension (maybe even DOI-based?).

   Yes, this is worthwhile; sample paths can be remote or local, or whatever you want, as they are just attributes of the sample. Related, in fact, PEP already supports links to remote CSV files. This got me thinking that it could be neat to use a google sheet as a sample table... not

currently possible, though. Clarified in the text as suggested.

7. You mention "data-intensive biological researhc projects". In your existing workflows, can you give an example on what scale of data you have successfully packaged in a PEP (number of parameters? number of records?) - I guess the limitation might not be so much data size, but complexity, so I'd have welcomed some illustration here as a reader. I don't see a specific reason why PEPs could not work for very very large datasets (validation does not seem to look into data files), so that could be worth mentioning [see also below re. binary data]

   We routinely use them to process hundreds of samples, up to thousands of samples. We're using PEP to run projects with about 20,000 samples right now (and starting to run into a few issues that we're working through). These probably have on the order of dozen or dozens of attributes per sample. Validation only confirms that data files exist, since we're just validating the metadata of the project. Clarified in the text as suggested.

8. Modifier import: How to the tools resolve imports? Would URLs work?

   This is a good idea. We will look into adding this functionality, here: https://github.com/pepkit/peppy/issues/362

   As for resolving them, here is what we wrote: Imports is the way to link two project configuration files. The values in the imported files will be overridden by the corresponding entries in the current configuration file. Imports are recursive, so an imported file that imports another file is allowed; the imports are resolved in cascading order with the most distant imports happening first, so the closest configuration options override the more distant ones.

9. Example for sample modifier duplicate: "oldattr: newattr" is less accessible than the other more realistic examples. Please consider changing to an example one might see in practice. Maybe "input: genome_hg38_validated_2020" ?

   Ok, modified to use the genome example from the text.

10. Sample modifier derive: Isn't encoding paths just one (even if most common) example of how to use "derive"? Second sentence of the paragraph and first sentence on page 5 do not fit together.

    Yes, derive can be used for anything. We clarified the text.

11. Sample modifier derive: The "shell variable" feature is indeed intriguing, but presented too brief for all users to make sense. I suggest to use a shell variable in the Example, or provide a footnote to an online example.

    We have extended this example to explain how you can do this. It's a very powerful use case.

12. Validation

- I suggest to add links to example schemas.

  OK, we have moved up the link to the eido documentation and clarified the location of schema examples.

- Add a reference for JSON-schema, possibly even JSON-schema validation.

  Link added. I can't find a paper for this.

- The website hosts the schemas in YAML, but not all YAML files are valid JSON. Also most readers might not know that YAML is a superset of JSON - strongly suggest to explain.

  Clarified, thanks.

- Since the tutorials on the eido website are not shown, it would be great to somehwere see the output or results of a validation.

  This was due to a temporary bug we had with readthedocs, and all tutorials render correctly now. We are also working on a web server that would allow you to explore this type of validation interactively.

13. PEP implementations

- If peppy instantiaties in-memore objects, does it load all data into memory?

  It loads all metadata *into memory – but doesn't touch any actual data files. It's solely about loading Project Objects and Sample Objects, which are, yes, loaded into memory. Referring back to the above, I suppose this means at least the current implementations are intended to handle projects whose metadata is not too large for memory. There is no restriction on actual data size, though. It would in theory be possible to write a separately implementation of the PEP spec that did not load all metadata into memory. I guess this would be an alternative to peppy or pepr, but still using the PEP spec.*

- Other than SnakeMake, what tools do you (envision to) use peppy or pepr? Would drake or targets (https://wlandau.github.io/targets/) be suitable candidates? Could be worth mentioning some, to illustrate.

  Yes, these are good possibilities. We will reach out to them. We have now been discussing this idea with Will Landau (author of drake and targets), and have a few ideas for integration. Others we have discussed with are: Nextflow, Galaxy, RnBeads, CWL. We also have a series of pipelines that we have written that use it, such as PEPATAC and PEPPRO, and BiocProject provides a Bioconductor R interface to PEP. I guess I'm a little reluctant to mention these possibilities in the published paper, since they may never decide to adopt PEP. But, I have been reaching out to people and trying to spread the word. The publication is an attempt to increase awareness of PEP, which I hope will drive adoption. We also have a java version in the works, but it's not ready for primetime.

14. How can a tool identify that something is a PEP? Do I need to look into all YAML files and see if there is pep_version?

    I suppose this would be one way. The way I would do it is to simply use eido to validate the object, and then rely on JSON schema. If it validates against the basic PEP schema, it qualifies as a PEP.

Discussion

I find the discussion interesting and sound. However, I'd really like to learn more about the possibilities around PEP, as they might also build a stronger case for it's usefulness. I hope you see room in the article for this, so here are some questions in different directions. If you find some useful and worth discussing, they could also make the "future work" aspect of the discussion more concrete and thereby accessible to readers.

1. What about source and authorship metadata, and licenses? I *like* that you do not replicate such information, but especially with the mentioned data fetchers, this information should also be available, and is important metadata if PEPs are shared. It may be worth clarifying where you expect these kind of information to be stored.

   Typically we have just been including attributes like `user` in the project configuration file, but I like the idea of a dedicated authorship block. I have started an issue here for discussion and we will brainstorm about the best way to do this: https://github.com/pepkit/pepspec/issues/13

It does seem like an accessory to the original intent, which is more about the computability and sharability of the metadata; the authorship would span more than just this metadata, and belongs with a publication of some kind, so I'm not convinced in belongs in the PEP specification. But, it does make sense to have some authorship information on datasets independent of a publication as well, so in that sense, it would make sense to put this information in the project config file.

- Your call to use the common format is applaudable - was any PEP-supporting tool developed outside of your lab yet? How can/will you support the community building and standardisation efforts? I'm not saying that you have to, but maybe you can point out suitable initiatives or organisations who could facilitate going from specification to standard, if they exist.

  There are a few of us using PEP from different labs now, but we haven't gotten word out too far. Anyone using our PEPATAC or PEPPRO software is using PEP. We have started to accumulate a list of studies that have made at least some use of PEP in the documentation here: http://pep.databio.org/en/latest/pep_in_practice/

- Suggest to add a link or a reference to the data fetcher.

  Agreed, as also raised by reviewer 2 – we have clarified this section in the discussion. See response to reviewer 2 for further discussion on related tools and future directions.

- Do you have plans to support samples that are not stored in a tabular form, e.g., binary formats such as NetCDF? Or databases? I think it would be interesting to briefly discuss if and how PEPs could support such data, especially considering the limits of plain text file formats for huge datasets.

  Well, since PEP is only really about metadata, and not about actual data, you'd have to get to a huge project before you can't store it as a CSV. That is indeed possible, but I guess outside the scope of what I imagined, which is that this is useful for scientific projects where we're dealing with samples, each of which probably represents an experiment, so supporting into the thousands seems useful but billions less so. But, something related that has comp up is this: https://github.com/pepkit/peppy/issues/333.

  NetCDF, for example, is really about actual data – not about sample attributes. So these kinds of things you could use as the data files that your PEP samples pointed to – but that's outside the scope of what PEP is standardizing.

  Databases is another idea that's definitely come up, though – even for sample metadata, having it in a database could be very useful.

- Do PEPs have any specific requirements when they are stored in data repositories? How could data repositories take advantage of them? I think this could be a further lever to improve community uptake (immediate benefits for repository operators or users).

  No, PEPs are simply files, and so they could be stored in a repository as simple as using a flat file object or block storage, or perhaps loaded into a database. There are no specific requirements. Indeed, data repositories benefit in two ways: First, PEPs could simplify submission. If a data repository provided a schema and said, you should submit data according to this format, then eido could be used to ensure compliance and provide a user with automated format feedback. It also would help standardize the data within the repository. Finally, the repository could provide the data in PEP format, making the dat available in a format that is immediately useful for downstream analysis. We have added a new paragraph in the discussion about uptake by data repositories.

- Are PEPs suitable as outputs for workflow tools and pipelines, or only as data sources?

In principle, PEP has been designed to serve as the metadata source. That being said, we recognized the potential of PEP to structure the output of a workflow. As a part of that we are currently developing a Python software package, pipestat, that reads an output schema that can be used to define the results produced by a workflow. This results in a possibility to enrich the input PEP with the output information and pass to the downstream analysis tools. BiocProject is our first effor to take advantage of this. To sum up, this is an area for a near future development.

- Did you consider using yaml features for referring to other fields, namely alias nodes (https://yaml.org/spec/1.2/spec.l ?

  We have not considered alias nodes in the past, thanks for bringing it up. In fact, any yaml feature could already be used in a PEP, as long it's supported by the underlying python/R yaml parser. So, it is already possible for someone to use advanced features if there was a use case for it. I have just tested using alias nodes with PEP and it works out of the box.

  I think this is a powerful feature that could be useful for some project configurations and I will try to use it in the future. One thing I immediately thought of is the possibility of providing node anchors in an external file, which is imported into a project using the `import` project_modifier. Unforunately, these alias nodes cannot be referenced from the main project configuration file, since importing is not a native yaml construct. However, we could think about tweaking the system to allow cross-file alias nodes, which could be useful.

- Please make your own software citable by depositing to a suitable repository, (Software Heritage or Zenodo - see GigaScience guidelines (https://academic.oup.com/gigascience/pages/editorial_policies_and_reporting_sta adding information to READMEs and then citing the deposits and the respective version of the software in the paper. This should also allow you to consistently refer to the different tools (e.g., looper is only mentioned by name while eilo is mentioned with a URL to the docs).

  Thanks. We will release all the software published in this paper with appropriate DOIs at Zenodo as requested, which is also required by GigaScience. We don't usually set these long-term identifiers until the publication is accepted, though, since there might be a flurry of activity as we finalize things during the review process and it feels cleaner to make the release corresponding to the final version. We do archive the development versions on both GitHub and PyPI, though. For software like looper that is tangential to this particular publication, we'll clean up the consistency of the citations as well. Our process is to release these to Zenodo upon publication; so if the software is unpublished at the time then we will cite the PyPI repository, which also qualifies as a citable identifier.

  We also added `eido`, `pepr`, and `peppy` to scicrunch and bio.tools. The identifiers are included in the revised manuscript.

- Please clarify how your tool relates to the approach presented at http://reproducible-bioinformatics.org/ and the papers mentioned there. Is it a different level of abstraction? I'm not a biologist, so I'm happy if you tell me this is completely unrelated.

  This is about processing data (here processing meaning running commands on actual data). PEP has no concept of running actual commands on actual data. It's completely about structuring project metadata*, like descriptions of the samples. The point of PEP is to decouple these two things. So, you could organize your data files and record them in a CSV table, with one row per sample, and store that metadata as a PEP. You could then read that metadata into R or python, and then you can run your REproducilbe Bioinformatics Analysis using whatever system you want (Snakemake, CWL, drake, SeqBox (the one you mention here), or whatever). PEP doesn't know anything about these actual systems for running reproducible*

code. The advantage of PEP is that you can give me your PEP, and now I can run my own PEP-compatible process on it as well. So, to conclude, PEP is an abstraction layer above these workflow management tools.

- You refer to a preprint about your own abstraction layer for containerisation (16). I think it is worth extending a bit on how PEP it's tools can play together with Bulker, Docker, or virtual machines.

  I cited container tools to indicate the interest in reproducible computing environments in general, where there has been a lot of advance; so it was an attempt to contrast that existing work, with the area where much less work has been done, in the interface of metadata and tools, where PEP sits. So, I view these things as complementary in that they are both pieces of a bigger puzzle to improve interoperability and reproducibility and reusability in scientific computing resources – tools, environments, data, and metadata. But, they clearly address different issues, and of course, the use of PEP can live within a containerized world just the way any software or data can. So, I think nothing particularly special there, other than that they have a common goal but address different levels of that software stack.

- Do you know the units package in R? I think PEP project files would be a powerful method to properly describe units and property types for CSV files. If you have thoughts about expicit typing of variables, I'd like to see them in the paper.

  I was not familiar with this package, but thanks for sharing. It is a good idea to establish explicit typing of variables in PEPs, and in fact, this is included in the eido functionality. The way we approach this now is to piggyback on JSON-schema to enable this. You would write a JSON-schema describing the sample and project attributes that are required – and their types, and any other validatable conditions, like ranges, regexes, allowable values, etc – and then you can validate the PEP against that schema with eido. We added a sentence in the eido section to make clear that this is the point of eido.

- I would have been interested to learn more about the motivations or roads *not* taken, e.g., have a section in the specification why YAML, why CSV. Sort of like the "Rationale" of the spec, but from a developer perspective. I think these decisions points are important for the "reproducibility" of the design, and can showcase the strengths of your approach.

  We attempted to answer this question in the Rationale *section of the specification here: http://pep.databio.org/en/latest/rationale/. I have added a section to this to give some rationale for the specific format decisions you cite.*

Practical evaluation:

Shell

```
python3 -m venv .venv
source ./.venv/bin/activate
pip install wheel
pip install peppy
pip install eido
git clone https://github.com/pepkit/example_peps
cd example_peps/

eido validate -s http://schema.databio.org/pep/2.0.0.yaml example_basic/project_config.yaml

python
```

Python

```
import peppy
proj1 = peppy.Project("example_basic/project_config.yaml")
```

R

```
renv::init() # already installs pepr because discovered in the R files within example_peps
install.packages("pepr")

library("pepr")
p = Project(file = "example_peps/example_basic/project_config.yaml")
sampleTable(p)
config(p)
```

Further comments

The following notes are only indirectly connected with the paper, but since I looked at things I'd like to share:

- For me the how-to guides on website are empty: http://peppy.databio.org/en/latest/tutorial/

  We had a temporary bug (a breaking change upstream in nbconvert) that broke all our readthedocs renders of tutorials. This has been fixed, thanks for catching.

- It would be nice if the example projects list on the PEP spec website would have some kind of index - it's great that there are so many, but the directory names only tell so much.

  Thanks for the suggestion. Updated.

- The list of "real datasets" in the PEP docs is impressive, yet it would be more useful if there is a one-liner description and if it is tagged by used PEP version.

  Updated

- In the examples, I noticed that some project configuration files include full paths ("/scratch/...") which might be tricky for third parties to use.

  Thanks for pointing this out, but we have not been able to locate these examples. Everything in the example repository (https://github.com/pepkit/example_peps) uses relative paths. If you can point us to the examples in question it would be helpful.

Reviewer #2:

This paper describes a new approach to describing sample metadata in a way that facilitates portability and reuse across workflows and pipelines (PEP - Portable Encapsulated Projects). The authors provide a formal specification for PEP and implementations in both python and R. In addition, they provide a validation tool for checking PEP compliance.

The central idea in this paper is interesting and addresses a real problem in bioinformatics. Although we are making good progress in scientific reproducibility for both data analysis, with workflows, and data management, with FAIR data, the "data interface" in between lags behind. PEP is designed to address this issue.

> *Thank you for the positive feedback. We hope we can convince you to try PEP out in linking data to workflows!*

The paper is well written and clear on its intentions and specifications, but it does not provide an evaluation of PEP as an approach. How has PEP been evaluated and what has it been compared to? Who is using

PEP and what were they using before? PEP is a possible solution to a real problem, but the current version of the paper only hints at real examples of use.

This has proven challenging because there really isn't much existing that we could find that is comparable to PEP. The only related thing of which we're aware is the ISA-tab framework, which we cited and compared in the introduction. But even this is not really that comparable, because it uses binary formats as opposed to simple plain text formats that are universally readable. The main alternative is that people just use their own CSV file with a custom schema to describe their data, an individual one for each project and each tool. All PEP really is doing, is publishing a specification to establish a standard, along with some tools to read that. As a result, we don't really see a way to evaluate or compare very extensively.

One thing we can do in response to this is provide additional detail as to how it has been used. Therefore, we've added some new text in the discussion section that points out how we're using PEP in practice. We developed this primarily because we had a need for it in our own biological research, so there are a few years of accumulated studies that have used the model that provide real examples of use. One of the primary reasons we're trying to publish a paper is to raise awareness of PEP, which is still primarily used by only a small handful of labs.

In the discussion, the authors mention a tool to fetch and transform SRA and GEO data. There is no link to this tool in the paper and no details of which projects make use of it (although the code is available in the GitHub repository). A better description of this work and its evaluation would already improve the results. A broader discussion of the overhead involved in doing the conversion would also be useful. Collecting data from SRA and GEO are common activities. Galaxy, for example, has its own solution to this already. How does this solution compare?

Yes, this refers to geofetch, which is our tool for creating a PEP from GEO/SRA accessions. You're absolutely right that a full description of geofetch and a comparison to galaxy will be useful, but we didn't include further details for two reasons: First, we felt that it was not a core part of the PEP specification, and we intended this paper to be a standalone specification. Therefore, we thought it appropriate to just mention in passing as a possible use case, along with a few others. We felt that this was outside the scope of the paper to include details about this, because the point of the paper was to describe the PEP specification.

Second, the geofetch software is much less polished than the core PEP software we described here. In fact, we intend to eventually write a paper describing geofetch, but it still needs more work. The documentation is sparse. We've used it in-house for dozens of projects, and it's open source and publicly available, but it's not really documented or organized in a way that others can use it easily, at the moment. We wanted the formal specification to be published and available first, and then we could turn attention there.

There are several other tools that fit this same category: looper, our PEP job submitter, and caravel, which provides a GUI to project management of PEPs, BiocProject, which links PEPs to bioconductor core objects. These are tools that build on PEP, but aren't really core parts of it; hence, we feel they should be described elsewhere. This paper is, in our vision, the first of a series of papers we'll publish over the next few years that build on PEP – but we first need to get the formal specification out there into the community. This is why we elected to only describe the tools we've developed that are integral to the specfication: eido, which exists solely to validate the PEP specification, and peppy and pepr, which are implementations of PEP specification, and nothing more. To extend the paper further we thought would be too much and should be described elsewhere, but we did think it worth mentioning.

To address this, we revised this section of the discussion, adding a bit more details about these tools but making clear that we view these as future projects that build on the current

work. Please see the new discussion, which has been rewritten.

In the long term, having a common format and approach would be beneficial. The discussion would be improved by a more detailed description of a pathway towards that goal. Would the authors aim to convince data providers to serve PEP compliant data, for example? Would the authors attempt to tackle legacy workflows, or simply recommend using PEP from now on?

Thanks for this comment. We've now restructured the discussion around this idea. Our "pathway toward the goal" is now structured with a few points: First, continuing the above discussion about related tools, we describe some of these related tools we're working on now a "future directions" and part of the pathway, which is to create additional tools surrounding the core PEP infrastructure presented in this manuscript. Second, we discuss the challenge to adopt PEP in workflow engines, and a vision for data repositories. I think it would be too much to ask for legacy data to be re-annotated, but if we can convince data providers and tool developers to begin to offer more data and tools around this format, then we can achieve a critical mass where the community will be able to standardize.
