# Supplementary material for: Linking big biomedical datasets to modular analysis with Portable Encapsulated Projects
Source: Gigascience. 2021 Dec 6;10(12):giab077. doi: 10.1093/gigascience/giab077 (PMC8673555; doi:10.1093/gigascience/giab077)
Supplement: giab077_Response_to_Reviewer_Comments_Revision_1 [file giab077_response_to_reviewer_comments_revision_1.pdf]

Author's Response To Reviewer Comments

Close

Response to review has been uploaded in the Personal Cover for formatting.

Close
