# Supplementary material for: Linking big biomedical datasets to modular analysis with Portable Encapsulated Projects
Source: Gigascience. 2021 Dec 6;10(12):giab077. doi: 10.1093/gigascience/giab077 (PMC8673555; doi:10.1093/gigascience/giab077)
Supplement: giab077_Reviewer_1_Report_Original_Submission — Daniel NÃ¼st, Dipl-Geoinf. -- 1/8/2021 Reviewed [file giab077_reviewer_1_report_original_submission.pdf]

Reviewer Report

Title: Linking big biomedical datasets to modular analysis with Portable Encapsulated Projects

Version: Original Submission **Date:** 1/8/2021

Reviewer name: Daniel NÃ¼st, Dipl-Geoinf.

Reviewer Comments to Author:

The manuscript at hand presents the PEP specification and corresponding tools.
It is well written and understandable and I have no comments on the form or structure.
The visual presentation of the concepts in Figs. 1-3 is excellent.
The simplistic specification suppoted by open tools are very good and presented very well. The modifier approach is novel to me and indeed powerful.
Thank you for a well-written article and the opportunity to get to know a very interesting collection of tools. I have no expertise in biological data, but could follow the text and successfully tried your tools, also thanks to the extensive online documentation.
The presented software seems to be vetted very well through previous usage in real scientific workflows and connected to other relevant tools (CWL, Snakemake) instead of reinventing the wheel.
The article requires minor revisions (no additional experiments or implementations).
Introduction
1. "Data generation has outpaced analysis, making analysis ..." - I stumbled over this a bit, a new notion for me, maybe you can provide a reference?
2. The colours of "Fits natively" and "Must reshape" were hard to distinguish at first glance - maybe use both colours and different dashes?
3. What is PEP's relation to common format specifications for scientific data from other domains? https://www.researchobject.org/ or eLife's ERA come to mind, though they have a larger scope and thereby are probably harder to establish than your more focused approach. IMO worth discussing, but not critical.
4. The presented Python and R tools seem to be only for reading PEPs. Can you please clarify what you mean with "tools that read and process PEPs" - I think I'm missing an example how then data is loaded from the PEP and some analysis actually happens. If this is to extensive to include, maybe reference one in the various online documentations of the tools.
5. Fig. 1G seems to be not fully explained. I understand this subfigure casts a wide net, but I'd still suggest to have a paragraph explaining your understanding or examples of these possibilities.
- How are PEPs useful for meta-analysis? What are "summarizers"?
- How are the connected to databases?
- 4. above may serve as an example for "Analysis tools"?
Results
1. I think the decision for YAML and CSV as file formats and the provisiong of Python and R libraries to use PEPs is great (suitability of the chosen methods).
2. However, the rationales could be explained a bit more in the text, e.g., plain text formats are great for

versioning and preserveration, but also have their limits; R + Python represent XX percent of the used tools (with reference).
3. Data citation (giving credit) is an important part of open science practice. Do you plan to explicitly support or model these kinds of relations in you configuration files, especially with project modifiers and re-combination of PEPs?
4. Is it possible to add arbitrary new fields to the configuration as a tool or user, or will it make it invalid?
5. Page 3 "see documentation for further details" would be more accessible with a footnote pointing to the meant docs.
6. Re. the contents of a PEP: "such as paths to sources of data" - Can you please clarify is this includes remote data sources? Might be worth putting into the discussion, if you think that could be a worthwhile extension (maybe even DOI-based?).
7. You mention "data-intensive biological researhc projects". In your existing workflows, can you give an example on what scale of data you have successfully packaged in a PEP (number of parameters? number of records?) - I guess the limitation might not be so much data size, but complexity, so I'd have welcomed some illustration here as a reader. I don't see a specific reason why PEPs could not work for very very large datasets (validation does not seem to look into data files), so that could be worth mentioning [see also below re. binary data]
8. Modifier import: How to the tools resolve imports? Would URLs work?
9. Example for sample modifier duplicate: "oldattr: newattr" is less accessible than the other more realistic examples. Please consider changing to an example one might see in practice. Maybe "input: genome_hg38_validated_2020" ?
10. Sample modifier derive: Isn't encoding paths just one (even if most common) example of how to use "derive"? Second sentence of the paragraph and first sentence on page 5 do not fit together.
11. Sample modifier derive: The "shell variable" feature is indeed intriguing, but presented too brief for all users to make sense. I suggest to use a shell variable in the Example, or provide a footnote to an online example.
12. Validation
- I suggest to add links to example schemas.
- Add a reference for JSON-schema, possibly even JSON-schema validation.
- The website hosts the schemas in YAML, but not all YAML files are valid JSON. Also most readers might not know that YAML is a superset of JSON - strongly suggest to explain.
- Since the tutorials on the eido website are not shown, it would be great to somehwere see the output or results of a validation.
13. PEP implementations
- If peppy instantiaties in-memore objects, does it load all data into memory?
- Other than SnakeMake, what tools do you (envision to) use peppy or pepr? Would drake or targets (https://wlandau.github.io/targets/) be suitable candidates? Could be worth mentioning some, to illustrate.
14. How can a tool identify that something is a PEP? Do I need to look into all YAML files and see if there is pep_version?
Discussion
I find the discussion interesting and sound. However, I'd really like to learn more about the possibilities

around PEP, as they might also build a stronger case for it's usefulness. I hope you see room in the article for this, so here are some questions in different directions. If you find some useful and worth discussing, they could also make the "future work" aspect of the discussion more concrete and thereby accessible to readers.
1. What about source and authorship metadata, and licenses? I _like_ that you do not replicate such information, but especially with the mentioned data fetchers, this information should also be available, and is important metadata if PEPs are shared. It may be worth clarifying where you expect these kind of information to be stored.
- Your call to use the common format is applaudable - was any PEP-supporting tool developed outside of your lab yet? How can/will you support the community building and standardisation efforts? I'm not saying that you have to, but maybe you can point out suitable initiatives or organisations who could facilitate going from specification to standard, if they exist.
- Suggest to add a link or a reference to the data fetcher.
- Do you have plans to support samples that are not stored in a tabular form, e.g., binary formats such as NetCDF? Or databases? I think it would be interesting to briefly discuss if and how PEPs could support such data, especially considering the limits of plain text file formats for huge datasets.
- Do PEPs have any specific requirements when they are stored in data repositories? How could data repositories take advantage of them? I think this could be a further lever to improve community uptake (immediate benefits for repository operators or users).
- Are PEPs suitable as outputs for workflow tools and pipelines, or only as data sources?
- Did you consider using yaml features for referring to other fields, namely alias nodes (https://yaml.org/spec/1.2/spec.html#id2786196) ?
- Please make your own software citable by depositing to a suitable repository, (Software Heritage or Zenodo - see GigaScience guidelines (https://academic.oup.com/gigascience/pages/editorial_policies_and_reporting_standards#Availability) , adding information to READMEs and then citing the deposits and the respective version of the software in the paper. This should also allow you to consistently refer to the different tools (e.g., looper is only mentioned by name while eilo is mentioned with a URL to the docs).
- Please clarify how your tool relates to the approach presented at http://reproducible-bioinformatics.org/ and the papers mentioned there. Is it a different level of abstraction? I'm not a biologist, so I'm happy if you tell me this is completely unrelated.
- You refer to a preprint about your own abstraction layer for containerisation (16). I think it is worth extending a bit on how PEP it's tools can play together with Bulker, Docker, or virtual machines.
- Do you know the units package in R? I think PEP project files would be a powerful method to properly describe units and property types for CSV files. If you have thoughts about expicit typing of variables, I'd like to see them in the paper.
- I would have been interested to learn more about the motivations or roads _not_ taken, e.g., have a section in the specification why YAML, why CSV. Sort of like the "Rationale" of the spec, but from a developer perspective. I think these decisions points are important for the "reproducibility" of the design, and can showcase the strengths of your approach.
Practical evaluation:
```bash
```

python3 -m venv .venv
source ./.venv/bin/activate
pip install wheel
pip install peppy
pip install eido
git clone https://github.com/pepkit/example_peps
cd example_peps/
eido validate -s http://schema.databio.org/pep/2.0.0.yaml example_basic/project_config.yaml
python
```
```Python
import peppy
proj1 = peppy.Project("example_basic/project_config.yaml")
```
```R
renv::init() # already installs pepr because discovered in the R files within example_peps
install.packages("pepr")
library("pepr")
p = Project(file = "example_peps/example_basic/project_config.yaml")
sampleTable(p)
config(p)
```
Further comments
The following notes are only indirectly connected with the paper, but since I looked at things I'd like to share:
- For me the how-to guides on website are empty: http://peppy.databio.org/en/latest/tutorial/
- It would be nice if the example projects list on the PEP spec website would have some kind of index - it's great that there are so many, but the directory names only tell so much.
- The list of "real datasets" in the PEP docs is impressive, yet it would be more useful if there is a one-liner description and if it is tagged by used PEP version.
- In the examples, I noticed that some project configuration files include full paths ("/scratch/...") which might be tricky for third parties to use.

Level of Interest

Please indicate how interesting you found the manuscript: Choose an item.

Quality of Written English

Please indicate the quality of language in the manuscript: Choose an item.
```

Declaration of Competing Interests

Please complete a declaration of competing interests, considering the following questions:

- Have you in the past five years received reimbursements, fees, funding, or salary from an organisation that may in any way gain or lose financially from the publication of this manuscript, either now or in the future?
- Do you hold any stocks or shares in an organisation that may in any way gain or lose financially from the publication of this manuscript, either now or in the future?
- Do you hold or are you currently applying for any patents relating to the content of the manuscript?
- Have you received reimbursements, fees, funding, or salary from an organization that holds or has applied for patents relating to the content of the manuscript?
- Do you have any other financial competing interests?
- Do you have any non-financial competing interests in relation to this paper?

If you can answer no to all of the above, write 'I declare that I have no competing interests' below. If your reply is yes to any, please give details below.

I declare that I have no competing interests

I agree to the open peer review policy of the journal. I understand that my name will be included on my report to the authors and, if the manuscript is accepted for publication, my named report including any attachments I upload will be posted on the website along with the authors' responses. I agree for my report to be made available under an Open Access Creative Commons CC-BY license (http://creativecommons.org/licenses/by/4.0/). I understand that any comments which I do not wish to be included in my named report can be included as confidential comments to the editors, which will not be published.

Choose an item.

To further support our reviewers, we have joined with Publons, where you can gain additional credit to further highlight your hard work (see: https://publons.com/journal/530/gigascience). On publication of this paper, your review will be automatically added to Publons, you can then choose whether or not to claim your Publons credit. I understand this statement.

Yes Choose an item.
