# Supplementary material for: Linking big biomedical datasets to modular analysis with Portable Encapsulated Projects
Source: Gigascience. 2021 Dec 6;10(12):giab077. doi: 10.1093/gigascience/giab077 (PMC8673555; doi:10.1093/gigascience/giab077)
Supplement: giab077_Reviewer_2_Report_Original_Submission — Katy Wolstencroft -- 1/18/2021 Reviewed [file giab077_reviewer_2_report_original_submission.pdf]

Reviewer Report

Title: Linking big biomedical datasets to modular analysis with Portable Encapsulated Projects

Version: Original Submission **Date:** 1/18/2021

Reviewer name: Katy Wolstencroft

Reviewer Comments to Author:

This paper describes a new approach to describing sample metadata in a way that facilitates portability and reuse across workflows and pipelines (PEP - Portable Encapsulated Projects). The authors provide a formal specification for PEP and implementations in both python and R. In addition, they provide a validation tool for checking PEP compliance.

The central idea in this paper is interesting and addresses a real problem in bioinformatics. Although we are making good progress in scientific reproducibility for both data analysis, with workflows, and data management, with FAIR data, the "data interface" in between lags behind. PEP is designed to address this issue.

The paper is well written and clear on its intentions and specifications, but it does not provide an evaluation of PEP as an approach. How has PEP been evaluated and what has it been compared to? Who is using PEP and what were they using before? PEP is a possible solution to a real problem, but the current version of the paper only hints at real examples of use.

In the discussion, the authors mention a tool to fetch and transform SRA and GEO data. There is no link to this tool in the paper and no details of which projects make use of it (although the code is available in the GitHub repository). A better description of this work and its evaluation would already improve the results. A broader discussion of the overhead involved in doing the conversion would also be useful. Collecting data from SRA and GEO are common activities. Galaxy, for example, has its own solution to this already. How does this solution compare?

In the long term, having a common format and approach would be beneficial. The discussion would be improved by a more detailed description of a pathway towards that goal. Would the authors aim to convince data providers to serve PEP compliant data, for example? Would the authors attempt to tackle legacy workflows, or simply recommend using PEP from now on?

Level of Interest

Please indicate how interesting you found the manuscript: Choose an item.

Quality of Written English

Please indicate the quality of language in the manuscript: Choose an item.

Declaration of Competing Interests

Please complete a declaration of competing interests, considering the following questions:

- Have you in the past five years received reimbursements, fees, funding, or salary from an organisation that may in any way gain or lose financially from the publication of this manuscript, either now or in the future?
- Do you hold any stocks or shares in an organisation that may in any way gain or lose financially from the publication of this manuscript, either now or in the future?
- Do you hold or are you currently applying for any patents relating to the content of the manuscript?
- Have you received reimbursements, fees, funding, or salary from an organization that holds or has applied for patents relating to the content of the manuscript?
- Do you have any other financial competing interests?
- Do you have any non-financial competing interests in relation to this paper?

If you can answer no to all of the above, write 'I declare that I have no competing interests' below. If your reply is yes to any, please give details below.

I declare that I have no competing interests

I agree to the open peer review policy of the journal. I understand that my name will be included on my report to the authors and, if the manuscript is accepted for publication, my named report including any attachments I upload will be posted on the website along with the authors' responses. I agree for my report to be made available under an Open Access Creative Commons CC-BY license (http://creativecommons.org/licenses/by/4.0/). I understand that any comments which I do not wish to be included in my named report can be included as confidential comments to the editors, which will not be published.

Choose an item.

To further support our reviewers, we have joined with Publons, where you can gain additional credit to further highlight your hard work (see: https://publons.com/journal/530/gigascience). On publication of this paper, your review will be automatically added to Publons, you can then choose whether or not to claim your Publons credit. I understand this statement.

Yes Choose an item.
