# Supplementary material for: Linking big biomedical datasets to modular analysis with Portable Encapsulated Projects
Source: Gigascience. 2021 Dec 6;10(12):giab077. doi: 10.1093/gigascience/giab077 (PMC8673555; doi:10.1093/gigascience/giab077)
Supplement: giab077_Reviewer_2_Report_Revision_1 — Katy Wolstencroft -- 6/7/2021 Reviewed [file giab077_reviewer_2_report_revision_1.pdf]

Reviewer Report

Title: Linking big biomedical datasets to modular analysis with Portable Encapsulated Projects

Version: Revision 1 **Date:** 6/7/2021

Reviewer name: Katy Wolstencroft

Reviewer Comments to Author:

This paper describes a new approach to describing sample metadata in a way that facilitates portability and reuse across workflows and pipelines (PEP - Portable Encapsulated Projects). The authors provide a formal specification for PEP and implementations in both python and R. In addition, they provide a validation tool for checking PEP compliance.
The ideas in this paper are good and the work addresses an important problem in bioinformatics. I think that PEP could be of benefit for managing the interface between data and analysis workflows, particularly across multiple workflow systems, but the revised version of the paper still does not present the evidence required to show this.
The latest version of this paper is written in a clear and logical way. It addresses some of the points from the previous review, but does not address the most important of those points. I asked who was using PEP, what it was being used for, and how it had been evaluated. For usage, the authors state that "We and others have successfully used this infrastructure in dozens of projects with hundreds to thousands of individual samples" Which projects, which samples and which workflows were run? At the very least, naming the projects would help here. Even better would be links to publications that use workflows to analyse data, where PEP formatting was used. In addition, are there available links to the PEP specifications created for this work?
For the evaluation, this point is not addressed. The paper would be much stronger if the authors could demonstrate that the introduction of the PEP format enabled easier workflow execution, or that specifying PEP format as input for a workflow enabled easier reuse of datasets.
The authors describe usage with Snakemake and CWL workflows as follows:
"Existing tools can easily accommodate the PEP structure; for example, SnakeMake includes a special directive to directly import a PEP into a workflow that functions alongside earlier, specialized data formats. Similarly, our companion tool, looper, can be used to submit arbitrary CWL workflows to a CWL runner for each sample in a PEP project. This sets the stage for a single data description that can be used as input for multiple workflows - even workflows built using different frameworks."
This sounds great, but it must be shown in the paper. Perhaps links could be provided to such CWL workflows and links to datasets that were run using them? If workflows from the workflow hub were used and data from public repositories, such as GEO, it would make a very powerful demonstration. One of the largest advantages of using workflows is that they can actually be executed.
In my review of the original article, I also asked what PEP could be compared to. In this version of the paper, PEP is compared to the ISA specification and the authors declare that PEP follows the goals of ISA. However, the ISA specification utilises community ontologies and controlled vocabularies for defining and describing metadata elements, particularly relating to biological sample attributes. In PEP,

there don't appear to be semantic descriptions, but simply name and value pairs. The semantic descriptions in ISA and other related metadata standard formats provide the ability to discover, understand and therefore reuse data. If PEP does not contain this rich semantic layer, is it intended to be provided in addition to existing metadata formats? If so, what is the incentive for data providers to increase their metadata burden? More importantly, if data is represented in a common metadata format, like ISA or MIAME etc, could it be automatically converted to a PEP specification?
How easy is it for a user to produce a PEP specification for their dataset and is that easier than simply writing a script to extract relevant columns and rows from a dataset that is already represented in a standard data format?
For figure 3, the diagrams are clear, but perhaps too simplistic to provide a concrete example. What is the difference between the generic and specific schemas? I would like to see a link to an actual generic and specific schema, so that readers can explore this in greater depth.

Level of Interest

Please indicate how interesting you found the manuscript: Choose an item.

Quality of Written English

Please indicate the quality of language in the manuscript: Choose an item.

Declaration of Competing Interests

Please complete a declaration of competing interests, considering the following questions:

- Have you in the past five years received reimbursements, fees, funding, or salary from an organisation that may in any way gain or lose financially from the publication of this manuscript, either now or in the future?
- Do you hold any stocks or shares in an organisation that may in any way gain or lose financially from the publication of this manuscript, either now or in the future?
- Do you hold or are you currently applying for any patents relating to the content of the manuscript?
- Have you received reimbursements, fees, funding, or salary from an organization that holds or has applied for patents relating to the content of the manuscript?
- Do you have any other financial competing interests?
- Do you have any non-financial competing interests in relation to this paper?

If you can answer no to all of the above, write 'I declare that I have no competing interests' below. If your reply is yes to any, please give details below.

I declare that I have no competing interests

I agree to the open peer review policy of the journal. I understand that my name will be included on my report to the authors and, if the manuscript is accepted for publication, my named report including any attachments I upload will be posted on the website along with the authors' responses. I agree for my report to be made available under an Open Access Creative Commons CC-BY license (http://creativecommons.org/licenses/by/4.0/). I understand that any comments which I do not wish to be included in my named report can be included as confidential comments to the editors, which will not be published.

Choose an item.

To further support our reviewers, we have joined with Publons, where you can gain additional credit to further highlight your hard work (see: https://publons.com/journal/530/gigascience). On publication of this paper, your review will be automatically added to Publons, you can then choose whether or not to claim your Publons credit. I understand this statement.

Yes Choose an item.
